# Post‐Synthetic Modification Unlocks a 2D‐to‐3D Switch in MOF Breathing Response: A Single‐Crystal‐Diffraction Mapping Study

**DOI:** 10.1002/anie.202105272

**Published:** 2021-07-12

**Authors:** Elliot J. Carrington, Stephen F. Dodsworth, Sandra van Meurs, Mark R. Warren, Lee Brammer

**Affiliations:** ^1^ Department of Chemistry University of Sheffield Brook Hill Sheffield S3 7HF UK; ^2^ Diamond Light Source Harwell Science and Innovation Campus Didcot OX11 0DE UK

**Keywords:** anisotropy, breathing mode, metal–organic frameworks, post-synthetic modification, single crystals

## Abstract

Post‐synthetic modification (PSM) of the interpenetrated diamondoid metal–organic framework (Me_2_NH_2_)[In(BDC‐NH_2_)_2_] (BDC‐NH_2_=aminobenzenedicarboxylate) **SHF‐61** proceeds quantitatively in a single‐crystal‐to‐single‐crystal manner to yield the acetamide derivative (Me_2_NH_2_)[In(BDC‐NHC(O)Me)_2_] **SHF‐62**. Continuous breathing behaviour during activation/desolvation is retained upon PSM, but pore closing now leads to ring‐flipping to avert steric clash of amide methyl groups of the modified ligands. This triggers a reduction in the amplitude of the breathing deformation in the two dimensions associated with pore diameter, but a large increase in the third dimension associated with pore length. The MOF is thereby converted from predominantly 2D breathing (in **SHF‐61**) to a distinctly 3D breathing motion (in **SHF‐62**) indicating a decoupling of the pore‐width and pore‐length breathing motions. These breathing motions have been mapped by a series of single‐crystal diffraction studies.

## Graphical abstract

Post‐synthetic modification of diamondoid MOF **SHF‐61** generates its methylamide analogue [(Me_2_NH_2_)[In(BDC‐NH(CO)Me)], **SHF‐62**, as single crystals. Resulting ligand steric demands and H‐bonding capability lead to a change in dimensionality of the MOF breathing behaviour from 2D breathing (pore width) to 3D breathing (pore width and length), which has been mapped by single‐crystal X‐ray diffraction.

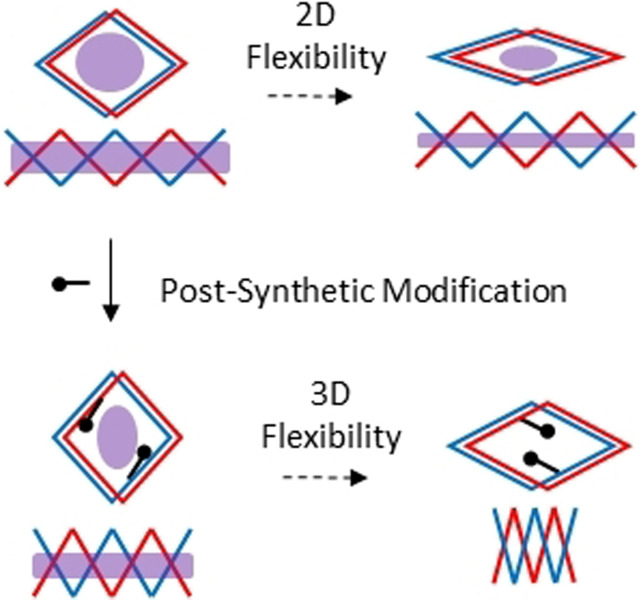

Metal–organic frameworks (MOFs), a class of porous coordination polymers, have attracted much research interest over the last two decades due to the wide range of potential applications afforded by their structural diversity, modular construction, chemical and spatial tunability and large internal surface areas.[^1^, ^2^, ^3^, ^4^, ^5^] A small subset of known MOFs, currently estimated at 0.1–1 %,[^6^, ^7^] exhibit significant dynamic structural responses,^[8]^ for example breathing or swelling behaviour during guest adsorption or release,[^9^, ^10^, ^11^, ^12^, ^13^, ^14^, ^15^, ^16^] and have been shown to offer further versatility including potential for improved performance in applications such as molecular sensing, separation, catalysis and drug delivery.[^17^, ^18^, ^19^, ^20^] Previous work in this area has shown that the use of different pendant functional groups on linker ligands is a promising method for fine‐tuning of the flexible behaviour with applications in mind.[^21^, ^22^, ^23^] Where direct incorporation of pendant groups via synthesis using substituted ligands has not been possible, post‐synthetic modification (PSM) methods have been employed to introduce these functionalities to an existing framework. For example, PSM of the framework DMOF‐NH_2_ enables control of the magnitude of its flexibility, while retaining the mode of flexibility.^[24]^ This ability to modulate the flexibility of MOFs, however, has only been shown for a limited number of framework materials which typically show stepwise breathing behaviours. The characterization of post‐synthetic modifications is often achieved through destructive techniques and consequently provides limited detail of the structural changes caused by addition of the new functional group. Relatively few PSM reports involve a single‐crystal‐to‐single‐crystal (SC‐SC) process, which enables more detailed structural analysis.[^25^, ^26^, ^27^]

We have previously reported the large‐amplitude 2D breathing behaviour and prominent host‐guest chemistry leading to CO_2_/CH_4_ gas separation capabilities of the diamondoid MOF (Me_2_NH_2_)[In(BDC‐NH_2_)_2_] (**SHF‐61**).[^28^, ^29^] A recent report has extended the potential application to a wider range of hydrocarbons.^[30]^ Here we report the post‐synthetic modification of **SHF‐61** by reaction with acetic anhydride at 55 °C in CHCl_3_ to yield the fully acetamide‐modified MOF (Me_2_NH_2_)[In(BDC‐NHC(O)Me)_2_] (**SHF‐62**). This occurs in a SC‐SC transformation and has a dramatic effect on the flexibility of the framework. **SHF‐61** was shown to exhibit a continuous and solvent‐dependent flexibility which, in contrast to almost all other known flexible frameworks, allows it to adopt a continuum of structures between wide‐pore and narrow‐pore forms, depending on the amount or type of solvent contained. This continuous “breathing” motion occurs predominantly via changes in the two dimensions perpendicular to its channels, whereas only much smaller changes occur along the channel direction. **SHF‐62** undergoes framework breathing via a similar mechanism during guest removal, but owing to the different intermolecular interactions between the two interpenetrated networks the relative magnitudes of framework motion are markedly different and substantial flexibility of the MOF is now observed in all three dimensions. The mechanistic details that give rise to the highly anisotropic breathing response to PSM have been mapped by single‐crystal X‐ray diffraction and clearly demonstrate the role of the modified functional group in triggering the new behaviour.

The structure of **SHF‐62**, in its solvated forms as **SHF‐62‐CHCl_3_** and **SHF‐62‐DMF**, was determined by single‐crystal X‐ray diffraction, facilitated by the SC‐SC nature of the PSM reaction in CHCl_3_ and subsequent solvent exchange to introduce DMF. The doubly‐interpenetrated anionic diamondoid network found in **SHF‐61** is maintained during the reaction, along with the lozenge‐shaped channels, which run along the *a*‐axis and contain dimethylammonium cations to balance the framework charge. The addition of the acetamide functional group, however, results in a 180° flip of the aromatic ring of the terephthalate ligand (the PSM‐flip), replacing the 4‐membered hydrogen‐bonding motif between amino groups observed in **SHF‐61** with a carbonyl‐carbonyl interaction between amides of the two interpenetrated networks (Figure 1). An accompanying small contraction of the framework along the channel length (Δ*a*≈−0.6 Å) provides an optimal geometry for this interaction and involves compression of the framework helices which run along the channel direction. Consistent with observations for the amine group of **SHF‐61**, the post‐synthetically added amide group in **SHF‐62** does not exhibit site disorder and is crystallographically characterized at full site occupancy, exclusively in one of the four sites on the aromatic ring. This ordering of the substituents is unusual in MOFs containing substituted terephthalate linkers, but here is consistent with the prominent role of the substituents in linking the two interpenetrated networks and is of consequence in the anisotropic modification of the framework breathing behaviour that results from PSM (vide infra).

**Figure 1:**
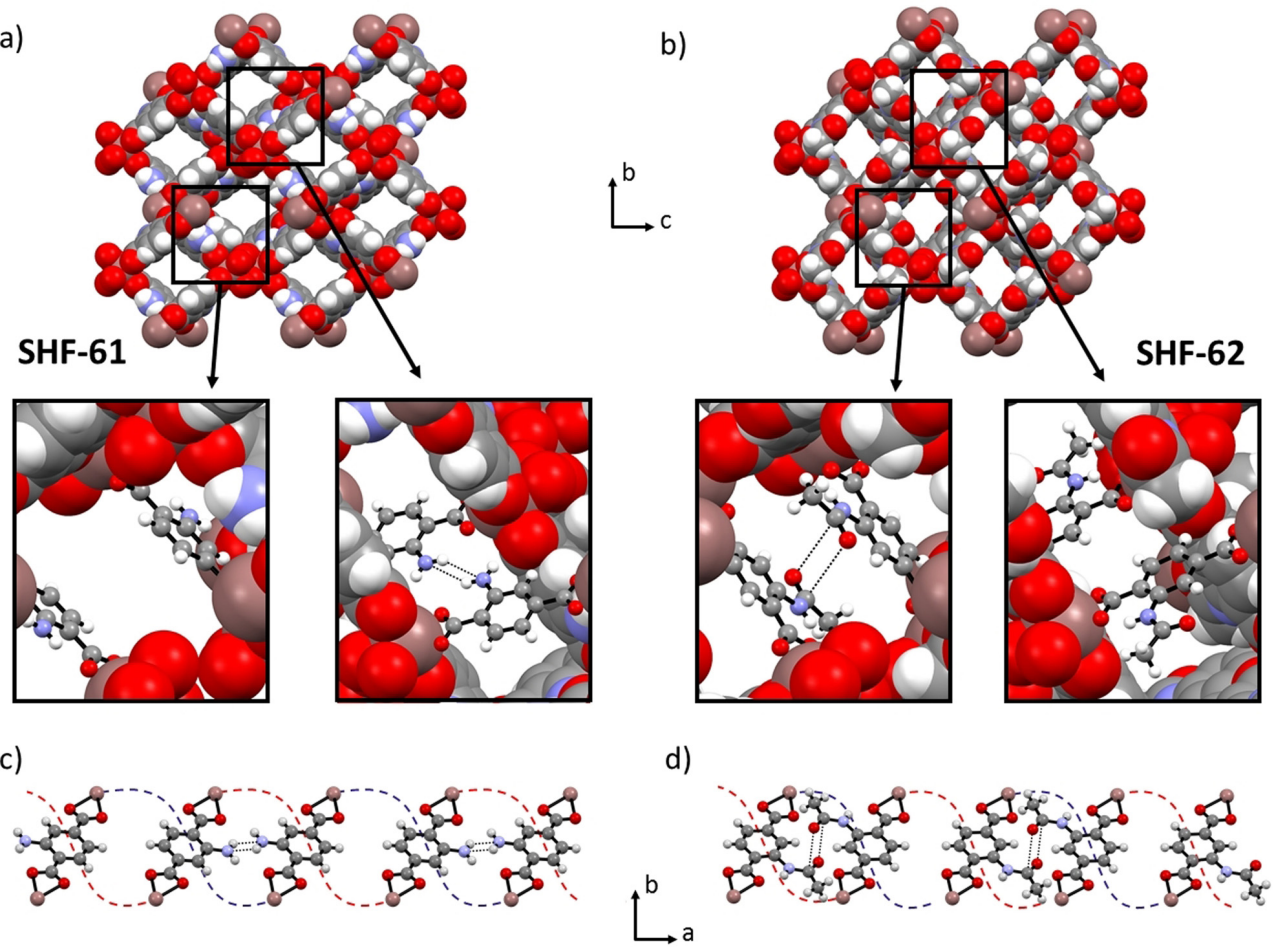
a) and b) View down the *a*‐axis showing the lozenge shape channels in the doubly‐interpenetrated frameworks of solvated **SHF‐61** and **SHF‐62**, respectively. Inset images show the relevant inter‐network interactions observed in the two crystal structures. c) and d) View down the *c*‐axis of **SHF‐61** and **SHF‐62**, respectively, showing the alternation along the *a*‐axis of the orientation of the arene substituent, leading to an alternating pattern of the inter‐network interactions (NH_2_⋅⋅⋅NH_2_ in **SHF‐61**, C=O⋅⋅⋅C=O in **SHF‐62**) and the absence thereof, occurring between the two interpenetrated networks. Red and blue dotted lines indicate the path of the two different interpenetrated networks which form helical chains that run along the channels in the *a*‐axis direction. The ring‐flip arising from PSM is evident from comparison of the structures of the two MOFs, that is, comparing insets in (a) with (b) or comparing (c) with (d). For full crystal structure data, see Ref. [31].

In contrast to the **SHF‐61** framework, the dimethylammonium cations are also localized within the pores of **SHF‐62** and could be characterized crystallographically. A hydrogen‐bonding interaction between the N‐H group of the cation and the carbonyl oxygen of the amide functionality is clearly identified. By contrast, in **SHF‐61** the amino group lone pair is sterically blocked by the other interpenetrated network, preventing such strong interactions with the cations. Guest CHCl_3_ molecules were also located crystallographically in **SHF‐62‐CHCl_3_**, and occupy the centre of the pore space. The CHCl_3_ guests can be readily exchanged for DMF molecules, which were identified in **SHF‐62‐DMF** by single‐crystal diffraction, ^1^H NMR spectroscopy and elemental analysis (see the Supporting Information).

The solvated forms of **SHF‐62** exhibit concerted differences in the *b*‐ and *c*‐axis lengths when containing different guests. Both **SHF‐62‐CHCl_3_** and **SHF‐62‐DMF** adopt the wide‐pore form, but **SHF‐62‐DMF** has more contracted pores (smaller *b*‐ and larger *c*‐axis lengths). Both have similar pore lengths (*a*‐axes). The behaviour of the framework during removal of the solvent molecules (i.e. activation) was mapped crystallographically in an analogous manner to that used for **SHF‐61**. Thus, a series of single crystals were each heated in situ using a nitrogen stream or ex situ using a temperature‐controlled oven, then returned to room temperature after fixed time periods prior to unit cell determination or full data collection at different stages of activation. Additional data were collected at 100 K in some cases (see the Supporting Information for details; see Figure 2).

**Figure 2:**
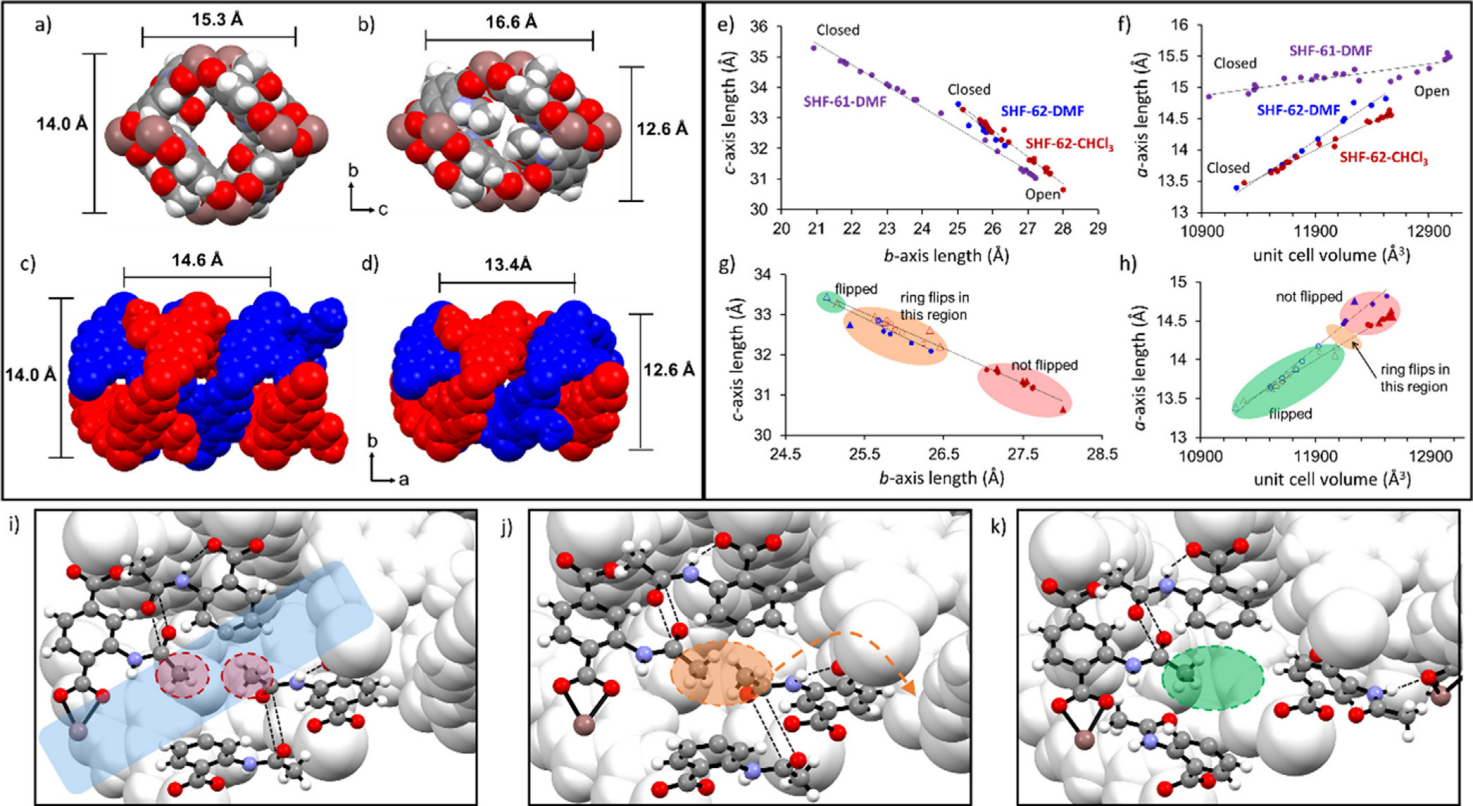
a) View down the *a*‐axis of **SHF‐62‐CHCl_3_** showing the lozenge‐shaped channel. b) View down the *a*‐axis of **SHF‐62** after solvent removal showing reduced channel area and congestion of the channels due to the blocking amide methyl groups. c) View down the *c*‐axis showing the helical arrangement of the two interpenetrated networks in **SHF‐62‐CHCl_3_**. d) View down the *c*‐axis for **SHF‐62** after solvent removal. e) Changes in the crystallographic *b*‐ and *c*‐axes during a series of in situ and ex situ single‐crystal heating experiments for **SHF‐62‐CHCl_3_** (red) and **SHF‐62‐DMF** (blue), shown for comparison with analogous experiments for **SHF‐61‐DMF** (purple). f) Changes in the *a*‐axis and unit cell volume during the same in situ heating experiments (as used in Figure 2 e) for **SHF‐62‐CHCl_3_** (red) and **SHF‐62‐DMF** (blue), compared with **SHF‐61‐DMF** (purple). g) and h) Expanded version of Figures 2 e and f, showing structural changes in **SHF‐62‐CHCl_3_** (red) and **SHF‐62‐DMF** (blue) and highlighting the region in which the ligand ring‐flip (activation‐flip) occurs, enabling further contraction of the *a*‐axis. Open symbols represent flipped‐ring structures; triangles represent full structure determination; circles represent unit‐cell determination. i) Inter‐framework (CO⋅⋅⋅CO) interactions of amide groups in **SHF‐62‐CHCl_3_**; no steric clash between methyl groups in wide‐pore solvated form. j) Predicted interactions between amide groups in **SHF‐62** after solvent removal if ligand rotation (and site disorder) did not occur. Steric clash of methyl group arising from pore contraction and narrowing is highlighted in orange ellipse. k) Relative positions of the amide groups in **SHF‐62** after ligand rotation (and site disorder) occurs, showing steric clash of methyl groups is averted. In Figures 2 e and f “Open” and “Closed” refer to the most open‐pore and most closed‐pore structures, respectively, in the studies conducted; crystallographic data are recorded at either 298 K or 100 K. In Figure 2 i, the semi‐transparent blue rectangle indicates the MOF channel. In Figures 2 i–k, indium ions and carboxylate groups have been removed for clarity. In all crystal structure images cations and solvent molecules have been removed for clarity. For full crystal structure data, see Ref. [31].

Removal of solvent guests resulted in a closing of the framework pores. Similar to the parent MOF (**SHF‐61**), the continuous flexibility of **SHF‐62** involves a contraction along the crystallographic *b*‐axis and an elongation along the *c*‐axis, resulting in reduction in the pore cross‐section in a *pseudo*‐wine‐rack manner. The overall extent of this deformation is less than observed upon DMF removal from **SHF‐61‐DMF**, but notably is also accompanied by a much larger change in the 3^rd^ dimension (*a*‐axis) which compresses the helical chains running parallel to the framework channels (Figures 2 a–f). Although the maximum change in unit cell volume is similar for the two materials (Δ*V*≈−2100 and −1600 Å^3^ for **SHF‐61** and **SHF‐62**, respectively), the breathing deformations occur distinctly in three dimensions in **SHF‐62**, in contrast to the ostensibly 2D breathing in **SHF‐61**. More detailed analysis of the crystal structures obtained at different levels of activation/desolvation show that this compression of the helical chains along the *a*‐axis causes a steric clash between methyl groups from pairs of adjacent amide substituents on different networks. This results in 50 % of the ligands rotating by approx. 150° (the activation‐flip) about the ring‐to‐carboxylate C−C bonds to avoid the unfavourable interaction, largely reversing their PSM‐flip and forcing the corresponding amide groups to point into the channels of the framework instead. The activation‐flip is accompanied by a 120° torsional rotation of the amide group out of the ring plane, allowing this arrangement to be stabilised by formation of a new inter‐network N−H⋅⋅⋅O hydrogen bond. This ligand rotation is modelled as a 50:50 crystallographic disorder within the single‐crystal structures.^[32]^ Figures 2 i–k illustrate this behaviour sequentially, firstly showing two amide groups before activation/desolvation, where no steric clash is present (Figure 2 i). The predicted steric clash that results from activation/desolvation is then illustrated (Figure 2 j) and finally the positions after the resulting flip of 50 % of the ligands to avert the clash (Figure 2 k). This rotation for half of the ligands occurs part way along the breathing pathway associated with activation/desolvation and takes place within a narrow region of channel lengths (14.2 Å≤*a*≤14.4 Å; Figure 2 h), but within a rather broader range of channel cross‐sections (25.3 Å≤*b*≤26.5 Å; 32.0 Å≤*c*≤33.0 Å; Figure 2 g). The consequence of the activation‐flip is an increased contraction of the channel lengths not observed in the parent **SHF‐61** MOF. The resulting positioning of half of the amide groups into the channel causes the pores to become more congested, reduces the accessible void volume, and limits contraction of the pore cross‐section compared to that accessible in **SHF‐61**. Figures 2 a and b show the view down one pore before and after solvent removal, respectively.

The strikingly different behaviour between **SHF‐61** and **SHF‐62** is thought to arise due to the change in the interactions between the two interpenetrated networks that is a consequence of PSM. **SHF‐61** displays a 4‐membered hydrogen‐bond motif between amine groups on different networks,^[28]^ whereas the amide groups of **SHF‐62** interact via carbonyl‐carbonyl interactions, following a 180° ring‐flip (PSM‐flip) of the amidoaryl group and resulting in the helical chains of the framework being more contracted (Δ*a*≈0.6 Å) in the resting wide‐pore state of the solvated forms. Further differences are also evident on comparison of the dynamic response to activation by removal of different solvents. **SHF‐61** is observed to be in a wide‐pore form after removal of CHCl_3_ and narrow‐pore after removal of DMF, whereas removal of either solvent in **SHF‐62** resulted in a similar flexible response, albeit from slightly different starting points, but with similar end points (Figure 2 e–h). Most significant, however, is the change upon activation from a 2D breathing response in **SHF‐61** to a 3D breathing response in **SHF‐62** (activation‐flip). This clearly demonstrates that although the breathing motions that affect pore cross‐section (changes in *b*‐ and *c*‐axis dimensions) are largely coupled in a *pseudo*‐wine‐rack manner, these deformations are essentially decoupled from the breathing motion that affects pore length (changes in *a*‐axis).

The overall behaviour of the two MOFs is summarised in Scheme 1, and highlights the structural changes observed during the single‐crystal mapping studies. The ability to locate both the solvent and cation molecules in the pore of the as‐synthesized **SHF‐62** during crystallographic studies suggests that there are stronger interactions between the framework and the pore contents in **SHF‐62** than **SHF‐61** and may also contribute to the change in behaviour. Further exploration of different solvents and the effect of any water content would be needed to draw more detailed conclusions; these aspects are being actively explored alongside computational modelling. Recent studies of MOFs comprising deformable M(O_2_CR)_4_^*n*−^ nodes[^28^, ^30^, ^33^, ^34^] illustrate that these represent an emerging class of responsive dynamic MOFs with tuneable behaviour. The amenability to study in situ by single‐crystal diffraction provides a route to detailed characterisation and understanding of this behaviour as a platform to future applications.

**Scheme 1:**
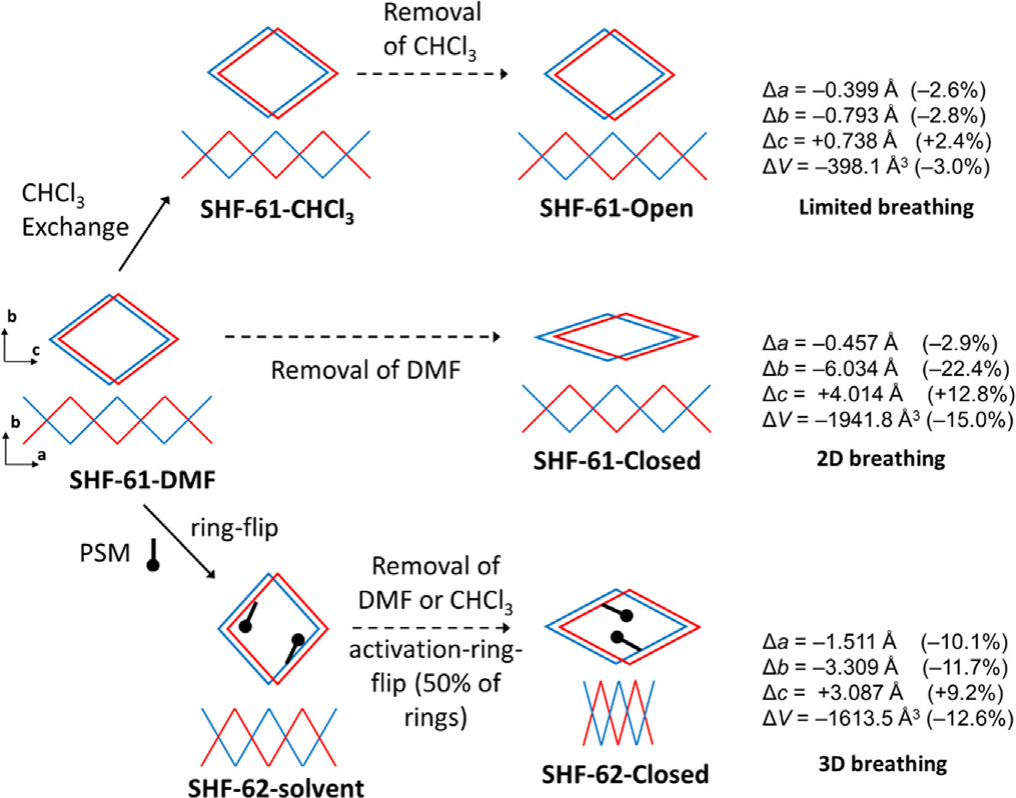
Representation of the different breathing behaviours of the MOFs **SHF‐61** and **SHF‐62** involving pore closing (**SHF‐61‐DMF**) and pore shortening (**SHF‐62‐solvent**) as a consequence of removal of solvent from the pores of the framework (activation).

## Conflict of interest

The authors declare no conflict of interest.

## Supporting information

As a service to our authors and readers, this journal provides supporting information supplied by the authors. Such materials are peer reviewed and may be re‐organized for online delivery, but are not copy‐edited or typeset. Technical support issues arising from supporting information (other than missing files) should be addressed to the authors.

Supporting Information
