# Supplementary material for: Post‐Synthetic Modification Unlocks a 2D‐to‐3D Switch in MOF Breathing Response: A Single‐Crystal‐Diffraction Mapping Study
Source: Angew Chem Int Ed Engl. 2021 Jul 12;60(33):17920–4. doi: 10.1002/anie.202105272 (PMC8457151; doi:10.1002/anie.202105272)
Supplement: Supplementary file 1 — Supporting Information [file ANIE-60-17920-s001.pdf]

Angewandte Chemie
Eine Zeitschrift der Gesellschaft Deutscher Chemiker

Supporting Information

Post-Synthetic Modification Unlocks a 2D-to-3D Switch in MOF Breathing Response: A Single-Crystal-Diffraction Mapping Study

*Elliot J. Carrington, Stephen F. Dodsworth, Sandra van Meurs, Mark R. Warren, and Lee Brammer**

anie_202105272_sm_miscellaneous_information.pdf

Table of Contents

1. Synthesis, Characterisation and Experimental Methods..S2
2. Crystallographic Characterisation of Post-Synthetic Modification S12
3. Crystallographic Characterisation of Activation and Breathing Behaviour....................................S16
4. Additional Graphical Representation of Breathing Behaviour S24
5. References...S25

1. Synthesis, Charaterisation and Experimental Methods

1.1 General

All reagents and solvents were purchased from Sigma-Aldrich or Alfa Aesar and were used without further purification, unless otherwise stated. Elemental analyses were recorded on a PerkinElmer 24000 CHNS/O Series II Elemental Analyser using combustion in pure oxygen. TGA data were obtained using a PerkinElmer Thermogravimetric Analyser in air.

1.2 Synthesis of $(Me_2NH_2)[In(BDC\text{-}NH_2)_2]\cdot xDMF\cdot yH_2O$ (SHF-61-DMF)

The parent MOF, **SHF-61-DMF**, was synthesised according to our previously reported procedure.[S1]

1.3 Synthesis of $(Me_2NH_2)[In(BDC\text{-}NH_2)_2]\cdot xCHCl_3\cdot yH_2O$ (SHF-61-$CHCl_3$)

The $CHCl_3$-exchanged parent MOF, **SHF-61-$CHCl_3$**, was synthesised as previously described.[S1]

1.4 Synthesis of $(Me_2NH_2)[In(BDC\text{-}NHC(O)CH_3)_{1.5}(BDC\text{-}NH_2)_{0.5}]\cdot CHCl_3$ (75% modification) (SHF-62-$CHCl_3$-PC)

Crystals of $(Me_2NH_2)[In(BDC\text{-}NH_2)_2]\cdot DMF$ (0.010 g, 0.02 mmol) were placed into 0.6 mL of HPLC grade $CHCl_3$ within a 1.5 mL screw-capped glass vial. 5 equivalents of acetic anhydride (10 μL, 0.1 mmol) were added and the vial was placed inside a temperature-controlled oven. The temperature was ramped to 55 ^oC at 2.5 ^oC/min and maintained for 24 hrs before cooling to room temperature at a rate of 0.1 ^oC/min. Upon removal from the oven the brown diamond-shaped crystals were still present and were transferred and stored in fresh $CHCl_3$.

1.5 Synthesis of $(Me_2NH_2)[In(BDC\text{-}NHC(O)CH_3)_2]\cdot CHCl_3$ (100% modification) (SHF-62-$CHCl_3$)

Crystals from $(Me_2NH_2)[In(BDC\text{-}NH_2)_2]\cdot DMF$ (0.100 g, 0.2 mmol) were placed into 0.6 mL of HPLC grade $CHCl_3$ within a 1.5 mL screw-capped glass vial. 5 equivalents of acetic anhydride (100 μL, 1 mmol) were added and the vial was placed inside a temperature-controlled oven. The temperature was ramped to 55 ^oC at 2.5 ^oC/min and maintained for 24 hrs before cooling to room temperature at a rate of 0.1 ^oC/min. Upon removal from the oven the brown diamond-shaped crystals were still present and were transferred and stored in fresh $CHCl_3$. Elemental analysis calculated for $(Me_2NH_2)[In(BDC\text{-}NHC(O)CH_3)_2]\cdot 0.95CHCl_3\cdot 1.85H_2O$: C, 36.75; H, 3.58; N, 5.60; Cl, 13.47. Found C, 36.74; H, 3.42; N, 5.51; Cl, 13.42.

1.6 Synthesis of $(Me_2NH_2)[In(BDC\text{-}NHC(O)CH_3)_2]\cdot DMF$ (SHF-62-DMF):

Single crystals of $(Me_2NH_2)[In(BDC\text{-}NHC(O)CH_3)_2]\cdot CHCl_3$ (approx. 0.100 g, 0.17 mmol) were placed in DMF (1 mL) for 1-2 weeks. DMF was replaced daily. Elemental analysis calculated for $(Me_2NH_2)[In(BDC\text{-}NHC(O)CH_3)_2]\cdot 1.15DMF\cdot 1.1H_2O$: C, 43.23; H, 4.60; N, 8.22. Found C, 43.22; H, 4.70; N, 8.17.

1.7 Thermogravimetric analysis of $(Me_2NH_2)[In(BDC\text{-}NHC(O)CH_3)_2]\cdot CHCl_3$ (SHF-62-CHCl$_3$)

TGA analysis was carried out on **SHF-62-CHCl$_3$** (5.4 mg) and **SHF-62-DMF** (3.6 mg) by heating the samples from 298 K (25 ^{o}C) to 973 K (700 ^{o}C) at a rate of 4 K/min. The samples were initially held at 298 K for 30 minutes before conducting the heating regime under flowing air (see Figures S1 and S2).

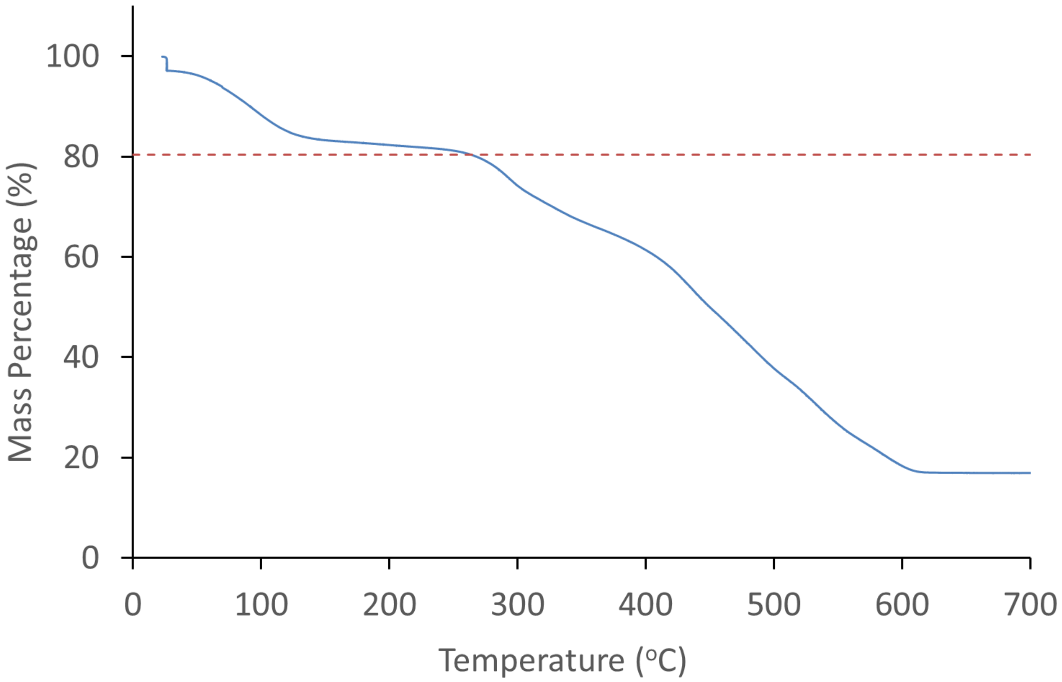

Figure S1. Thermogravimetric analysis of **SHF-62-CHCl$_3$** recorded at a ramp rate of 4 K/min. The dotted red line indicates the expected mass loss of the contained solvent calculated from elemental analysis.

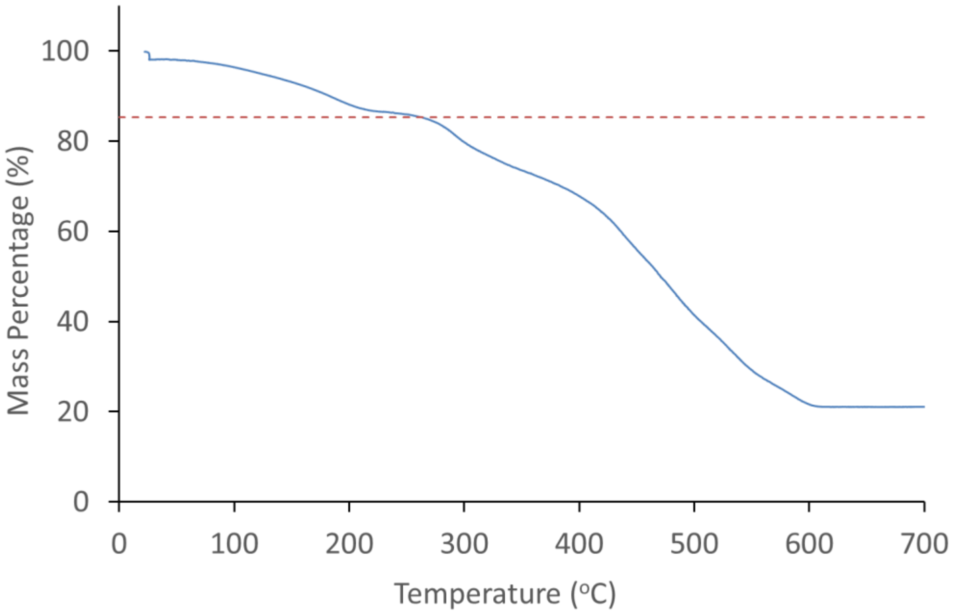

Figure S2. Thermogravimetric analysis of **SHF-62-DMF** recorded at a ramp rate of 4 K/min. The dotted red line indicates the expected mass loss of the contained solvent calculated from elemental analysis.

1.8 Solution-Phase ^{1}H NMR spectroscopy of digested SHF-62-CHCl$_3$

Solution-phase ^{1}H NMR spectroscopy (400 MHz, DMSO-d$_6$) was carried out using a Bruker DPX-400 spectrometer. The MOF sample **SHF-62-CHCl$_3$** (10 mg) was digested using 50 µL of acid (35% DCl in D_2O) in 1 mL of DMSO-d$_6$, and the spectrum recorded without neutralising the solution (Figure S3). All constituent parts were observed to be soluble in the DMSO after digestion. The full conversion from **SHF-61-DMF** was evident from the change in the shifts of the aromatic protons on the ligand, which was also analysed using the same method (Figure S4).

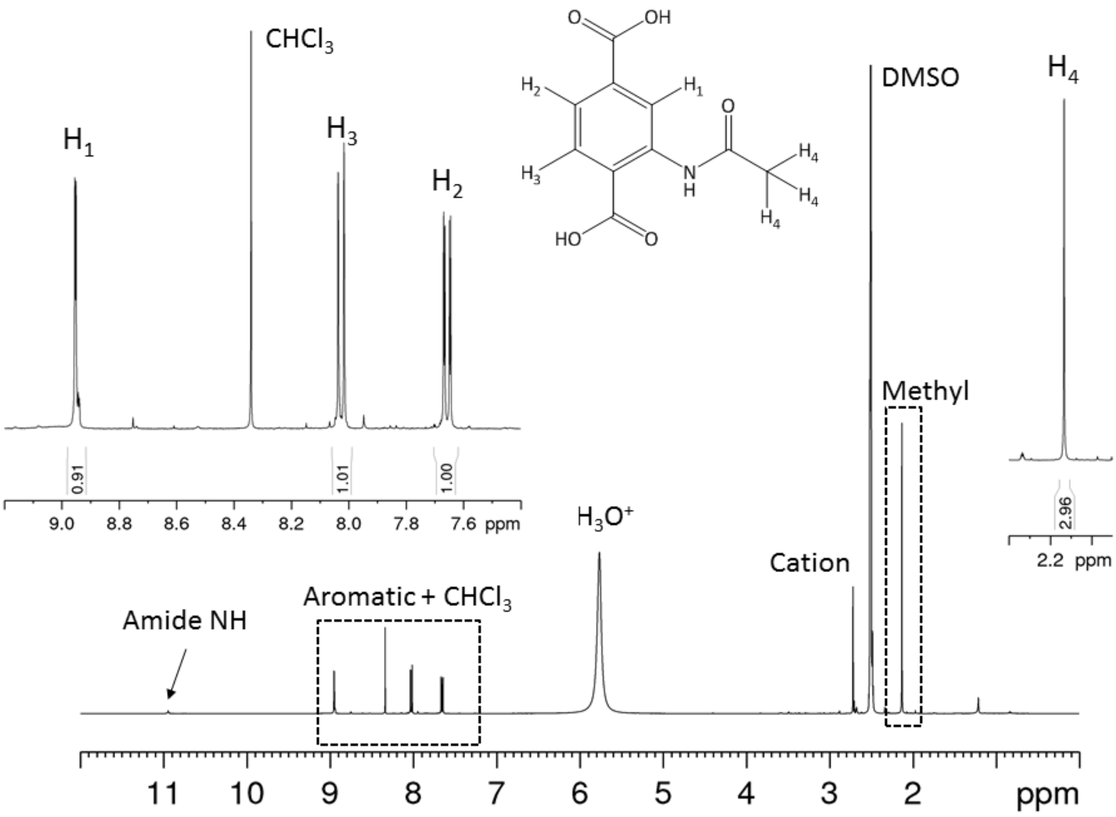

Figure S3. ^{1}H NMR spectrum measured at 298 K for digested **SHF-62-CHCl$_3$**

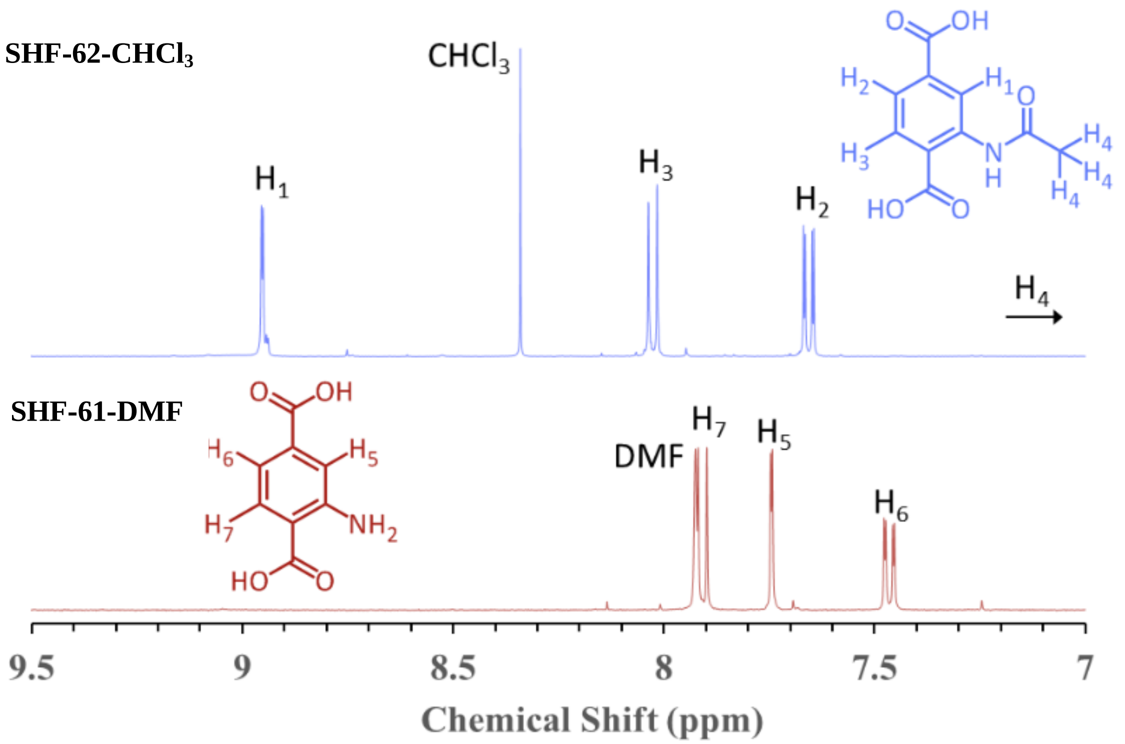

Figure S4. Comparison of the aromatic region of the ^1H NMR spectra measured at 298 K for both digested **SHF-62-$CHCl_3$** (blue) and digested **SHF-61-DMF** (red). The individual resonances have been assigned based on the J-coupling of signals.

The ^1H NMR spectrum of MOF sample **SHF-62-DMF** (5 mg) was recorded by following the same digestion procedure. The spectrum (Figure S5) shows that DMF has replaced $CHCl_3$ as the solvent, and that the ligand modification is maintained.

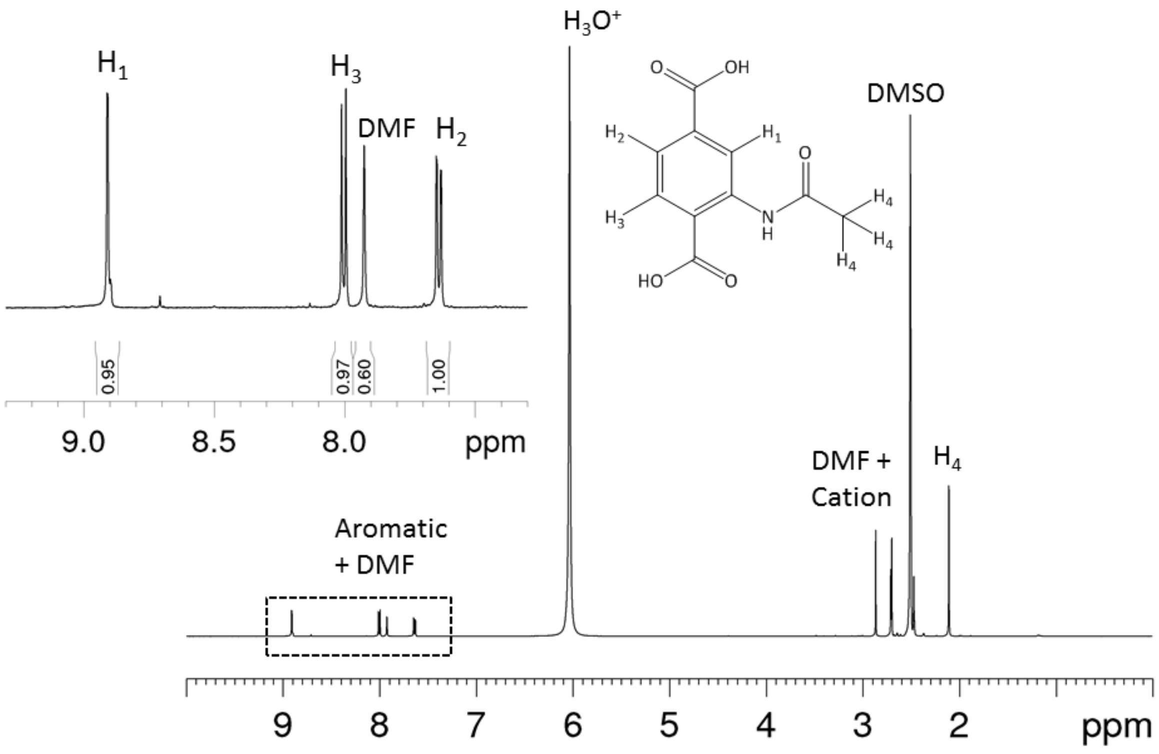

Figure S5. ^1H NMR spectrum measured at 298 K for digested **SHF-62-DMF**

^{1}H NMR spectroscopic data were also recorded on sample SHF-62-**$CHCl_3$-PC** using the same methodology. The conversion from **SHF-61-DMF** was judged to be 75-80% based on the relative integrations of the aromatic protons on the ligand. The spectrum is shown in Figure S6.

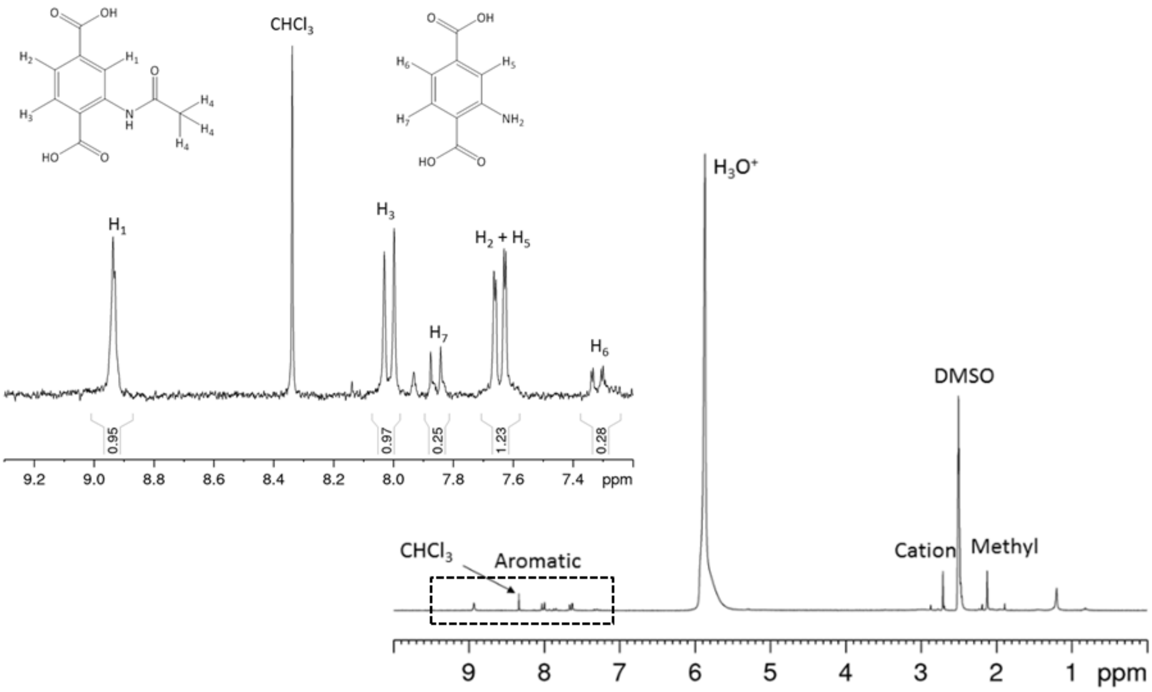

Figure S6. ^{1}H NMR spectra measured at 298 K for digested **SHF-62-$CHCl_3$-PC**.

1.9 Solid-state ^{13}C NMR spectroscopy

Solid-state ^{13}C NMR spectroscopy (500 MHz for ^{1}H) was carried out on **SHF-61-$CHCl_3$** and **SHF-62-$CHCl_3$** (Figure S7, Tables S1 and S2). Analogous characterisation for **SHF-61-DMF** has been previously reported.[S1] Samples (100 mg) were ground into a fine powder, packed into 4 mm zirconia rotors and transferred to a Bruker AVANCE III HD spectrometer. Pseudo-quantitative 1D ^{13}C (with high-power ^{1}H decoupling) magic-angle spinning (MAS) NMR experiments were performed at a MAS rate of 10.0 kHz. Spectra were measured using a relaxation delay of 300 s in approximation of quantitative conditions. (T1s for ^{13}C centres were not determined). Measurements were acquired until sufficient signal-to-noise was observed. Values of the chemical shifts are referenced to adamantane. The magnetic field was set to place the higher frequency ^{13}C (methylene) resonance of adamantane at a chemical shift of 38.48ppm. Integrals were obtained by deconvolution.

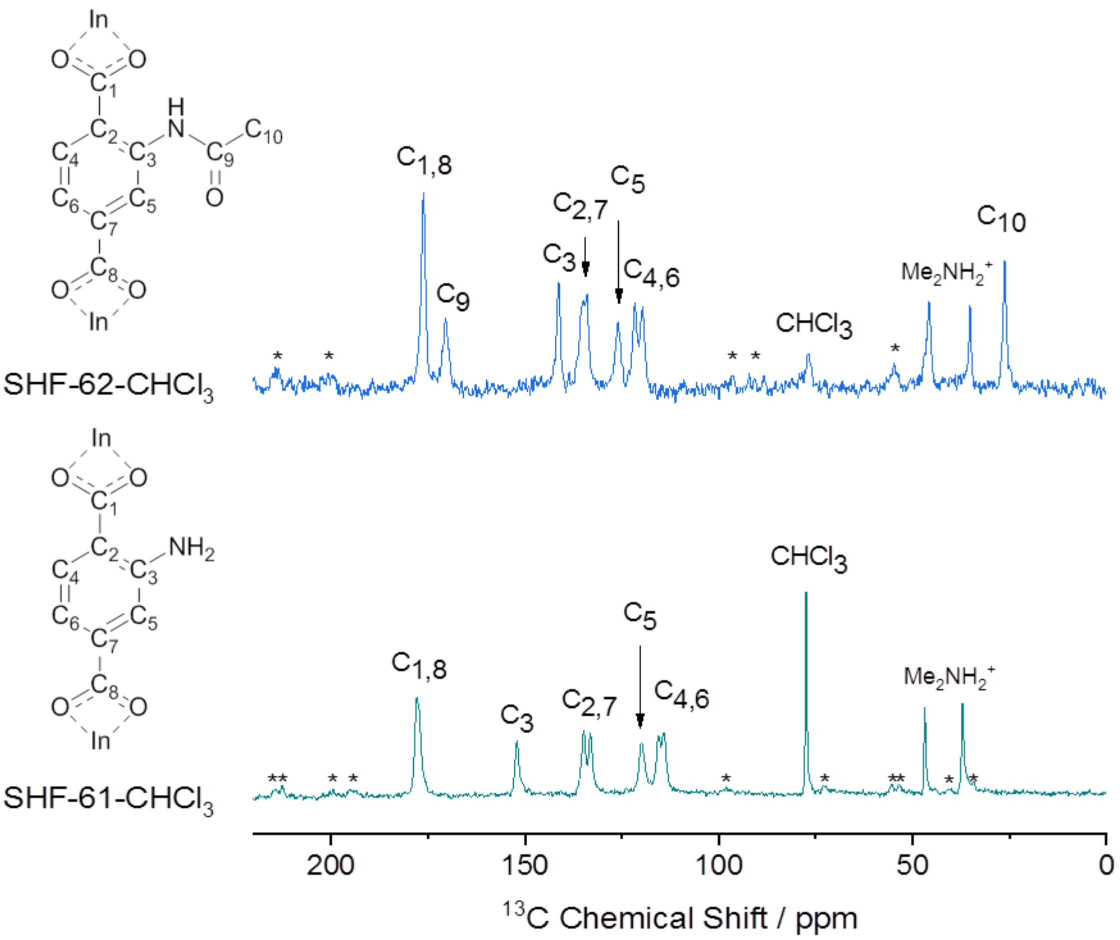

Figure S7. Normalised, solid-state quantitative ^{13}C NMR spectra recorded at 298 K for **SHF-61-CHCl$_3$** (green) and **SHF-62-CHCl$_3$** (blue). Asterisks are used to denote spinning sidebands.

Table S1. Chemical shifts, peak widths and areas and integration values obtained via deconvolution of ^{13}C signals for **SHF-61-CHCl$_3$**.

Assignment	Chemical Shift (ppm)	Peak Width (Hz)	Area	Integral
C_1,C_8	177.7	184.1	1968.7	2
C_3	152.1	138.8	755.3	0.77
C_2	134.8	163.6	1066.9	1.08
C_7	133.2	152.7	928.0	0.94
C_5	119.9	206.2	1063.4	1.08
C_4	115.5	201.9	1191.5	1.21
C_6	114.3	184.0	1174.5	1.19
$CHCl_3$	77.5	48.2	1023.0	1.03
$Me_2NH_2^+$	46.8	55.9	499.0	0.51

$Me_2NH_2^+$	37.1	87.6	783.2	0.80

Table S2. Chemical shifts, peak widths and areas and integration values obtained via deconvolution of ^{13}C signals for **SHF-62-CHCl$_3$**.

**Assignment**	**Chemical Shift (ppm)**	**Peak Width (Hz)**	**Area**	**Integral**
C_1,C_8	176.2	146.5	3056.6	2.11
C_9	170.5	187.7	1329.8	0.91
C_3	141.4	129.2	1446.0	1.0
C_2,C_7	134.7	277.1	2819.0	1.95
C_5	126.0	186.0	1251.0	0.86
C_4	121.6	179.8	1567.8	1.08
C_6	119.8	155.6	1365.1	0.94
$CHCl_3$	76.9	170.0	603.8	0.41
$Me_2NH_2^+$	45.9	158.4	1369.6	0.94
$Me_2NH_2^+$	35.2	86.5	728.4	0.50
C_{10}	26.3	115.5	1523.4	1.05

1.10 Solid-state ^{15}N NMR spectroscopy

Solid-state ^{15}N NMR spectroscopy (500 MHz for ^1H) was carried out on **SHF-61-CHCl$_3$**, **SHF-61-DMF**, **SHF-62-CHCl$_3$** and an activated sample of **SHF-62-CHCl$_3$** (Figure S8, Table S3). Activation was achieved by heating a sample of **SHF-62-CHCl$_3$** in a Schlenk tube at 353 K for 16 hours under high vacuum. Samples (100 mg) were ground into a fine powder, packed into 4 mm zirconia rotors and transferred to a Bruker AVANCE III HD spectrometer. 1D ^1H-^{15}N cross-polarisation magic-angle spinning (CP-MAS) NMR experiments were performed at a MAS rate of 10.0 kHz. Spectra were recorded using a contact time of 2.0 ms. The relaxation delay D_1 for each sample was individually determined from the proton T_1 measurement (D_1 = 5 x T_1). Measurements were acquired until sufficient signal-to-noise was observed. Values of the chemical shifts are referenced to adamantane. The magnetic field was set to place the higher frequency ^{13}C (methylene) resonance of adamantane at a chemical shift of 38.48ppm.

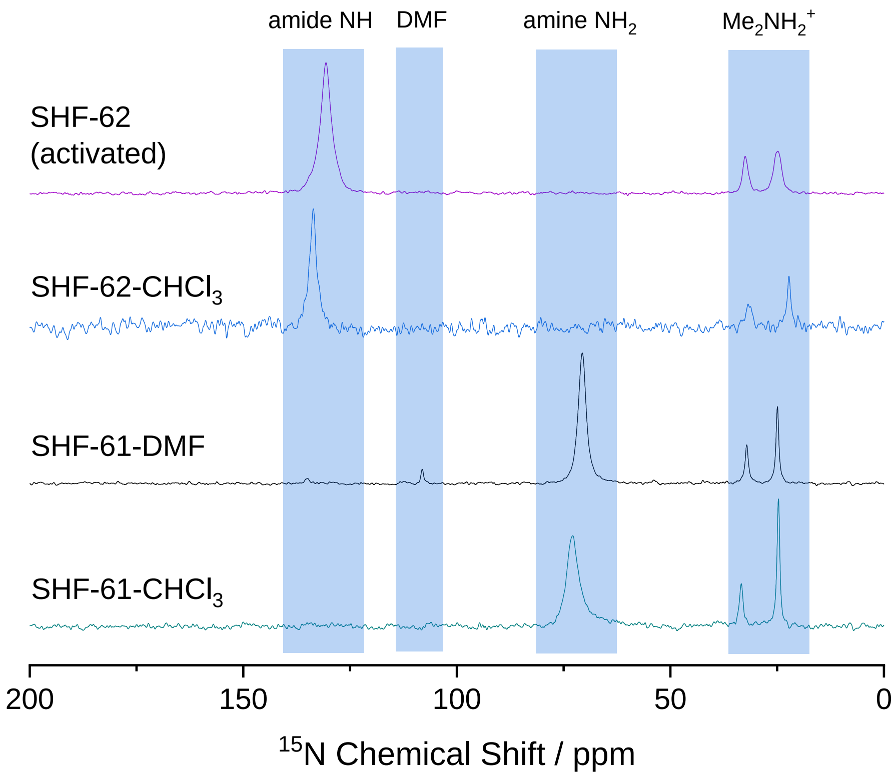

Figure S8. Solid-State ^{15}N NMR spectra recorded at 298 K for **SHF-61-CHCl$_3$** (green), **SHF-61-DMF** (black), **SHF-62-CHCl$_3$** (blue) and activated **SHF-62** (purple)

Table S3. ^{15}N chemical shifts for **SHF-61-CHCl$_3$**, **SHF-61-DMF**, **SHF-62-CHCl$_3$** and activated **SHF-62**.

**Compound**	**Chemical Shift (ppm)**		
	**Amine substituent**	**Amide substituent**	**Me$_2$NH$_2$$^+$**
**SHF-61-CHCl$_3$**	72.83	–	33.38, 24.68
**SHF-61-DMF**	70.61	–	32.11, 24.92
**SHF-62-CHCl$_3$**	–	133.59	31.80, 22.22
**SHF-62** (activated from **SHF-62-CHCl$_3$**)	–	130.63	32.47, 24.82

1.11 Solid-state IR spectroscopy

Solid-state IR spectra were recorded between 4000 cm^{-1} and 500 cm^{-1} for **SHF-62-DMF**, **SHF-62-CHCl$_3$** and **SHF-61-DMF** using a PerkinElmer Spectrum 100 spectrometer fitted with a universal diamond ATR accessory. The spectra are shown in Figure S9.

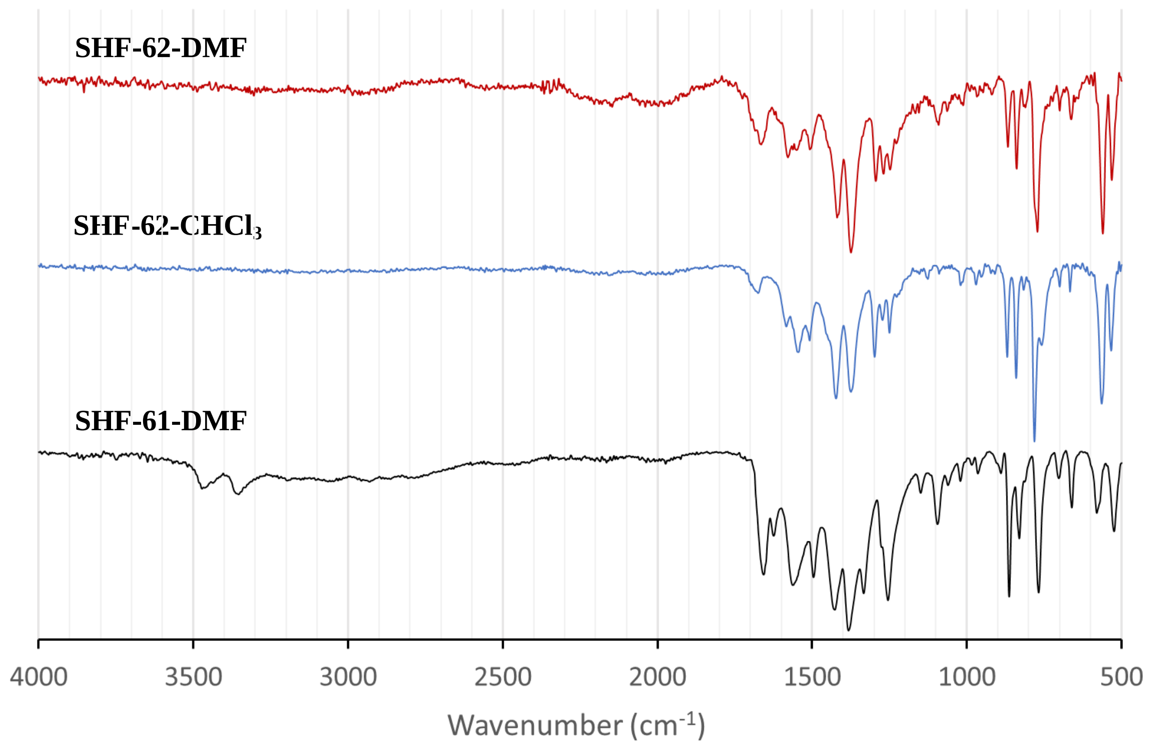

Figure S9. Solid-state IR spectra measured at 298 K for **SHF-62-DMF** (red), **SHF-62-$CHCl_3$** (blue) and **SHF-61-DM**F (black).

1.12 Single-Crystal X-Ray Diffraction

Laboratory single-crystal X-ray diffraction data were collected on a Bruker *SMART APEX-II* CCD diffractometer operating a Mo-K_α sealed-tube X-ray source or a Bruker D8 Venture diffractometer equipped with a *PHOTON* 100 dual-CMOS chip detector and operating a Cu-K_α IμS microfocus X-ray source. The data were processed using either the *APEX2*[S2] software or the *CrysAlisPro* Software.[S3] X-Ray data were corrected for absorption using empirical methods based upon symmetry-equivalent reflections combined with measurements at different azimuthal angles (*SADABS*).[S4] An Oxford Cryosystems Cryostream device was used to maintain the sample temperature. Synchrotron single-crystal X-ray diffraction data were collected at beamline I19 (EH1), Diamond Light Source.[S5] Data were collected at a wavelength of 0.6889(1) Å using a Fluid Film Devices Ltd diffractometer equipped with a *PILATUS* 2M detector. Sample temperature was controlled using an Oxford Cryosystems Cryostream Plus device. Data were processed using DIALS software[S6] and corrected for absorption using empirical methods (*SADABS*).[S4]

All crystal structures were solved and refined against F^2 values using the program *SHELXL*[S7] accessed within the *OLEX2* program.[S8] All non-hydrogen atoms were refined anisotropically except for some of the structures in which positional disorder of the cations or orientational disorder of the linker ligand was required. Crystallographic restraints and constraints were applied to some structures where necessary. Positions of hydrogen atoms were calculated with idealised geometries and refined using a riding model with isotropic displacement parameters. The *PLATON* function *SQUEEZE*[S9] was applied

in some cases to determine the contribution to the structure factors of the unmodelled electron density of solvent molecules and/or the cations in the MOF pores, where otherwise poor convergence of the least-squares refinement resulted. As an illustrative example, for the crystal structure refinement of **SHF-62-$CHCl_3$-PC** (Section 2), *SQUEEZE* was used to treat the cation and solvent molecules as a contribution to the overall scattering of diffuse electron density without modelling specific atom positions, but cation and guest solvent molecules were able to be modelled for **SHF-62-$CHCl_3$** and **SHF-62-DMF**. The calculation by *SQUEEZE* on **SHF-62-$CHCl_3$-PC** indicated that the unit cell contains 1432 electrons in the void, corresponding to approximately one cation molecule and one $CHCl_3$ molecule per formula unit. These values are reasonable and compare well to **SHF-62-$CHCl_3$** where one cation molecule and 0.5 $CHCl_3$ molecules per formula unit were able to be modelled crystallographically. For crystal structure refinements described in section 3 analogous approach was adopted in only where needed. Full details are available in the CIFs, which were checked using *checkCIF*/*PLATON*,[S10] and include responses to any alerts.

1.13 Powder X-Ray Diffraction

Laboratory powder diffraction data were obtained using a Bruker D8 Advance powder diffractometer equipped with focusing Göbel mirrors, recorded in the range $3\ ^{\circ} \leq 2\theta \leq 50\ ^{\circ}$, using Cu-$K_{\alpha}$ radiation. Data were collected in a Debye-Scherrer geometry with rotating capillary stage and samples loaded in 0.7 mm borosilicate capillaries.

Synchrotron powder diffraction data were collected at beamline I11 at Diamond Light Source using a wide-angle (90 °) position sensitive detector (PSD) comprising 18 Mythen-2 modules.[S13, S14] A pair of scans related by a 0.25 ° detector offset was collected for each measurement to account for gaps between detector modules. The resulting patterns were summed to give the final pattern for analysis. Powdered samples were loaded into 0.7 mm borosilicate capillaries. Indexing, Pawley[S13] and Rietveld[S14] refinements were carried out using *TOPAS* Academic version 4.1.[S15] Indices of fit between the calculated and experimental diffraction patterns (R_{wp} and R_{wp}') are defined by the equations below.

$$R_{wp} = \sqrt{\frac{\Sigma[w(Y_{\mathrm{obs}} - Y_{\mathrm{calc}})^2]}{\Sigma[wY_{\mathrm{obs}}{}^2]}}$$

Equation S1. Index R_{wp} used in powder diffraction fitting

$$R_{wp}{}' = \sqrt{\frac{\Sigma[w(Y_{\mathrm{obs}} - Y_{\mathrm{calc}})^2]}{\Sigma[w(Y_{\mathrm{obs}} - bkgr)^2]}}$$

Equation S2. Index R_{wp}' used in powder diffraction fitting

2. Crystallographic Characterisation of Post-Synthetic Modification

2.1 Crystal structures of SHF-62-$CHCl_3$ and SHF-62-DMF

Crystal structures of **SHF-62-$CHCl_3$, SHF-62-DMF** and **SHF-61-$CHCl_3$-PC** were obtained by transferring the single crystals obtained from the synthesis directly into a perfluoropolyether oil (FOMBLIN Y), mounting a suitable crystal onto a MiTeGen 200 μm MicroMount under an optical microscope, and transferring this to the diffractometer where the crystal was immersed in the dry nitrogen stream of the cryostream device at 100 K. Significant differences in unit cell dimensions are observed between the framework containing the two different solvents. The crystal data are shown in Table S4.

Table S4. Data collection, structure solution and refinement parameters for single crystal X-ray structure determinations of **1**.

	**SHF-62-$CHCl_3$ (A1)**	**SHF-62-DMF (A2)**	**SHF-62-$CHCl_3$-PC (A3)**
Crystal Habit	Octahedron	Octahedron	Octahedron
Crystal Colour	Brown	Brown	Brown
Crystal Size (mm)	0.19 × 0.17 × 0.11	0.16 × 0.12 × 0.06	0.14 × 0.13 × 0.1
Crystal System	Orthorhombic	Orthorhombic	Orthorhombic
Space Group, Z	*Fddd*, 16	*Fddd*, 16	*Fddd*, 16
a (Å)	14.633(3)	14.7591(6)	14.6266(7)
b (Å)	28.004(6)	25.3156 (11)	26.8907(12)
c (Å)	30.640(7)	32.7537(13)	31.5398(15)
α (°)	90	90	90
β (°)	90	90	90
γ (°)	90	90	90
V (Å^3)	12556(5)	12237.9(13)	12405.2(10)
Radiation	Cu-K_α (λ = 1.54178 Å)	Cu-K_α (λ = 1.54178 Å)	Mo-K_α (λ = 0.71073 Å)
Density (g cm^{-3})	1.420[c]	1.442[c]	1.243[b]
Temperature (K)	100	100	100
μ (mm^{-1})	7.660[c]	6.691[c]	0.806[b]
2θ Range (°)	7.40 to 133.48	7.44 to 133.502	3.98 to 54.96
Reflns collected	17599	18798	29284
Independent reflns (R_{int})	2780 (0.0293)	2720 (0.0766)	3554 (0.0602)
Reflns used in refinement, n	2780	2720	3553
L.S. parameters, p	169	172	153
No. of restraints, r	10	7	22
Completeness	0.996	0.997	0.998
$R1(F)$[a] $I > 2\sigma(I)$	0.0647	0.0560	0.0410
$wR2(F^2)$[a], all data	0.2069	0.1653	0.1301
$S(F^2)$[a], all data	1.098	1.058	1.061

[a] $R1(F) = \sum(|F_o| - |F_c|)/\sum|F_o|$; $wR2(F^2) = \sqrt{\sum w(F_o^2 - F_c^2)^2/\sum wF_o^4}$; $S(F^2) = \sqrt{\sum w(F_o^2 - F_c^2)^2/(n + r - p)}$.

[b] Density and adsorption coefficient are calculated using only framework atoms and cations, and do not include guest molecules.

[c] Density and adsorption coefficient are calculated using framework atoms and cations, and modelled guest molecules.

2.2 Bulk-phase analysis of SHF-62-$CHCl_3$ and SHF-62-DMF

The phase purity of **SHF-62-$CHCl_3$** was confirmed through X-ray powder diffraction. A room temperature pattern (**B1**) was collected on the laboratory instrument (Cu-Kα) as detailed on page S12. The unit cell parameters from the single-crystal structure of the as-synthesised MOF (**A1**) were used as starting point for a Pawley refinement, employing 311 parameters (10 background, 1 zero error, 5 profile, 3 cell and 292 reflections), resulting in final indices of fit R_{wp} = 4.337, $R_{wp'}$ = 8.132. The fit is shown in Figure S10. The final unit cell parameters were orthorhombic a = 14.8985 (3) Å, b = 28.3190 (7) Å, c = 30.3815 (8) Å, V = 12818.3 (5) Å^3.

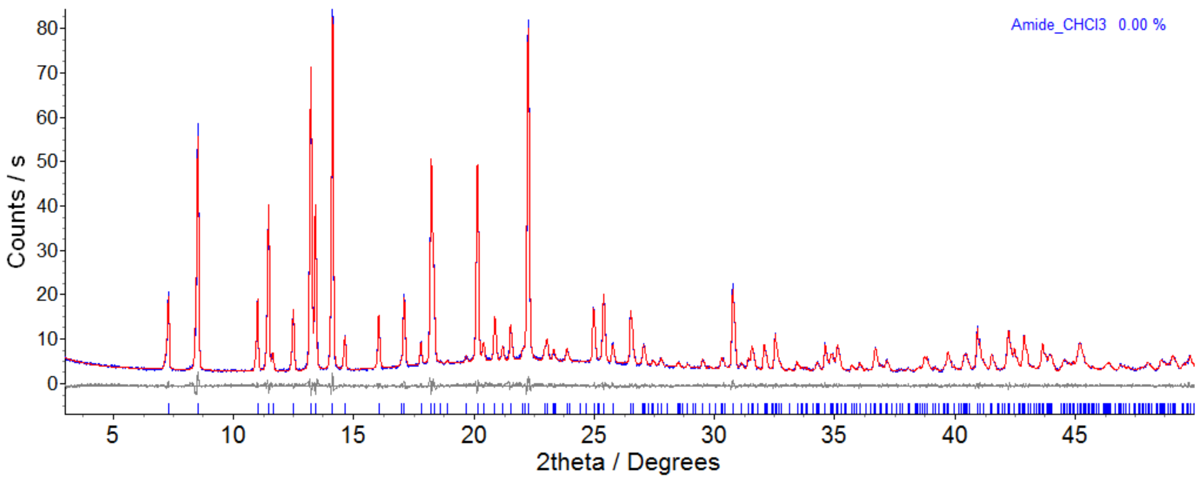

Figure S10. Observed (blue) and calculated (red) and difference plot [I_{obs}-I_{calc}] (grey) of the Pawley[S13] refinement of **SHF-62-$CHCl_3$** (**B1**) (2θ range 3.0 – 50.0 °, d_{min} = 1.8Å).

The phase purity of **SHF62-DMF** was also confirmed through X-ray powder diffraction. A high-resolution room-temperature pattern (**B2**) was collected at the I11 beamline[S11,S12] as detailed on page S12, λ = 0.826210(5) Å. The unit cell parameters from the single crystal structure of the as-synthesised MOF (**A2**) were used as starting point for a Pawley refinement, employing 1,855 parameters (10 background, 1 zero error, 5 profile, 3 cell and 1,836 reflections), resulting in final indices of fit R_{wp} = 3.412, $R_{wp'}$ = 10.855. The framework atoms of **A2** were then used as a starting point for a Rietveld refinement employing 21 parameters (10 background, 1 zero error, 5 profile, 3 cell, 1 scale, 1 occupancy), resulting in final indices of fit R_{wp} = 5.431, $R_{wp'}$ = 18.714. The fits are shown in Figures S11 and S12. The positions and orientations of the cation and solvent molecules (modelled as rigid bodies) were optimised by simulated annealing. The final unit cell parameters were orthorhombic a = 14.8774 (2) Å, b = 26.2123 (4) Å, c = 32.1573 (6) Å, V = 12540.4 (3) Å^3.

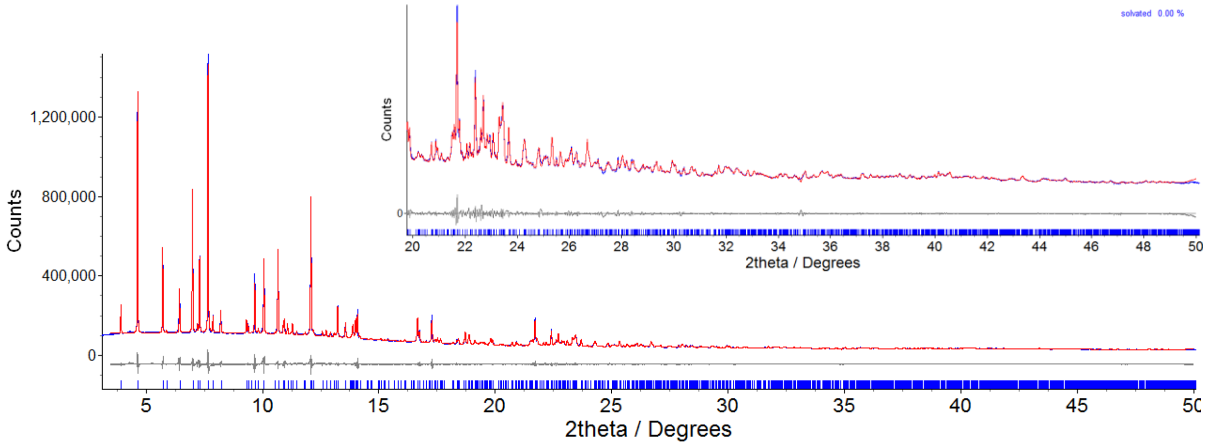

Figure S11. Observed (blue) and calculated (red) and difference plot [I_{obs}-I_{calc}] (grey) of the Pawley[S13] refinement of **SHF-62-DMF** (**B2**) (2θ range 3.0 – 50.0 °, d_{min} = 0.98Å).

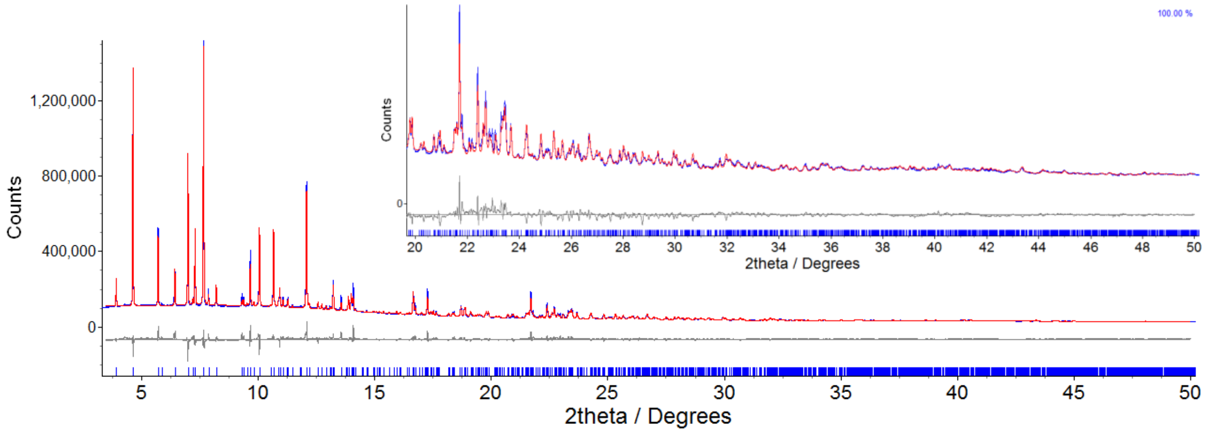

Figure S12. Observed (blue) and calculated (red) and difference plot [I_{obs}-I_{calc}] (grey) of the Rietveld[S14] refinement of **SHF-62-DMF** (**B2**) $(Me_2NH_2)[In(BDC\text{-}NHC(O)CH_3)_2]\cdot 0.47DMF$ (2θ range 3.0 – 50.0 °, d_{min} = 0.98Å).

3. Crystallographic Characterisation of Activation and Breathing Behaviour

3.1 Crystallographic studies of SHF-62-$CHCl_3$ following *in situ* heating

Crystals used for *in situ* heating were selected while immersed in the mother liquor and one face of the crystal glued to a glass fibre or MiTiGen MicroMount while the crystal was still covered in a thin layer of residual solvent. Care was taken to avoid coating the entire crystal in adhesive. The crystal was rapidly transferred to the diffractometer and situated in the nitrogen stream of an Oxford Cryosystems Cryostream device at room temperature. The glue was left to dry for 15 mins before any data were collected. Heating was carried out *in situ* using the Cryostream device. Seven different heating experiments were carried out on seven separate crystals of **SHF-62-$CHCl_3$** (**C1–C7**). Experiments **C1**–**C5** were performed using a lab diffractometer, as described in section 1.12. Experiments **C6** and **C7** were carried out at beamline I19, Diamond Light Source, as described in section 1.12.[S5] The crystals **C5–C7** were prepared using dry $CHCl_3$ and those in **C3** and **C4** were exchanged with dry $CHCl_3$ prior to the diffraction experiment ($CHCl_3$ dried according to the method of Grubbs[S16]).

A ramp rate of 4 K/min was used to raise the temperature, which was held at the final value for a set period (see Table S5) before cooling at the same ramp rate. Intensity data collections were recorded under the nitrogen stream at 298 K, using the Cryostream to maintain the temperature. In experiment **C3** the crystal was then cooled to 100 K using the Cryostream to observe the thermal effect on the flexibility. The unit cell parameters for experiments **C1**–**C5** were obtained by analysing reflections with $I/\sigma > 10$ from four sets of 10 ^{o} omega scans with 0.5 ^{o} slicing. Full data collections were recorded at the end of studies **C1** (at 298 K), **C3** (at 100 K) and **C4** (at 298 K), due to the sample maintaining a high level of crystallinity (**C1.3**, **C3.3** & **C4.4**). A full data collection was also recorded for experiment **C5.2**. Full data collections were recorded throughout studies **C6** and **C7**. Table S5 details the seven separate heating studies carried out and the resulting cell parameters. Details of the full data collections are shown in Table S6.

Table S5. Parameters used and unit cell parameters determined for crystallographic studies of desolvation of **SHF-62-$CHCl_3$** by *in situ* heating. All data were collected at 298 K, except C3.3 which was recorded at 100 K.

Heating Study	Temp heated	Hold time at max. temp.	Data Set Code	*a* (Å)	*b* (Å)	*c* (Å)	*V* ($Å^3$)
**C1**	25 ^oC	Start	**C1.1**	14.518(5)	27.525(7)	31.264(6)	12493(6)
	80 ^oC	5 mins	**C1.2**	14.44(1)	27.18(1)	31.55(1)	12379(11)
	150 ^oC		**C1.3**	13.6309(3)	25.6303(7)	32.9437(7)	11509.4(5)
**C2**	25 ^oC	Start	**C2.1**	14.453(4)	27.039(9)	31.620(8)	12357(6)
	150 ^oC	5 mins	**C2.2**	13.80(2)	25.89(3)	32.65(3)	11670(20)
**C3**	25 ^oC	Start	**C3.1**	14.541(8)	27.63(2)	31.191(9)	12532(10)
	150 ^oC	15 mins	**C3.2**	13.654(9)	25.78(2)	32.860(9)	11565(10)
	Cooled to 100K		**C3.3**	13.469(1)	25.153(2)	33.274(2)	11273.1(15)
**C4**	25 ^oC	Start	**C4.1**	14.573(5)	27.62(2)	31.160(9)	12542(9)
	150 ^oC	15 mins	**C4.2**	13.721(3)	25.83(1)	32.768(7)	11614(8)
	150 ^oC	15 mins	**C4.3**	13.710(3)	25.81(1)	32.796(7)	11606(7)
	175 ^oC	15 mins	**C4.4**	13.678(1)	25.747(1)	32.7999(9)	11551(12)
**C5**	25 ^oC	Start	**C5.1**	14.522(7)	27.513(13)	31.243(8)	12483(3)
	80 ^oC	5 mins	**C5.2**	14.174(1)	26.453(2)	32.207(2)	12076.1(13)
	80 ^oC	15 mins	**C5.3**	14.089(15)	26.245(19)	32.276(13)	11934(15)
	100 ^oC	5 mins	**C5.4**	13.882(14)	25.973(19)	32.540(12)	11733(14)
	100 ^oC	30 mins	**C5.5**	13.795(14)	25.88(2)	32.617(9)	11646(14)
**C6**	25 ^oC	Start	**C6.1**	14.5562(2)	27.5448(4)	31.3490(3)	12569.3(3)
	120 ^oC	15 mins	**C6.2**	14.0557(8)	26.3239(10)	32.6166(10)	12068.2(9)
**C7**	25 ^oC	Start	**C7.1**	14.5486(2)	27.4966(4)	31.3509(4)	12541.5(3)
	80 ^oC	5 mins	**C7.2**	14.4748(2)	27.1714(5)	31.6622(5)	12452.8(3)

Table S6. Data collection, structure solution and refinement parameters for crystal structures during activation (solvent removal) studies on **SHF-62-$CHCl_3$**

	In situ heating of **SHF-62-$CHCl_3$ (C1.3)**	*In situ* heating of **SHF-62-$CHCl_3$ (C3.3)**	*In situ* heating of **SHF-62-$CHCl_3$ (C4.4)**
Crystal Habit	Octahedron	Octahedron	Octahedron
Crystal Colour	Brown	Brown	Brown
Crystal Size (mm)	0.20 × 0.20 × 0.10	0.35 x 0.35 x 0.15	0.25 × 0.2 × 0.1
Crystal System	Orthorhombic	Orthorhombic	Orthorhombic
Space Group, Z	*Fddd*, 16	*Fddd*, 16	*Fddd*, 16
a (Å)	13.6309(3)	13.4693(11)	13.6778(12)
b (Å)	25.6303(7)	25.1528(18)	25.7472(12)
c (Å)	32.9437(7)	33.274(2)	32.7999(9)
α (°)	90	90	90
β (°)	90	90	90
γ (°)	90	90	90
V (Å^3)	11509.4(5)	11273.1(15)	11551.0(12)
Radiation	Cu-K_α (λ = 1.54178 Å)	Cu-K_α (λ = 1.54178 Å)	Cu-K_α (λ = 1.54178 Å)
Density (g cm^{-3})[b]	1.393	1.422	1.388
Temperature (K)	298	103	298
μ (mm^{-1})[b]	7.027	7.175	7.002
2θ Range (°)	10.74 to 133.19	7.91 to 118.06	7.80 to 117.84
Reflns collected	26635	12962	12815
Independent reflns (R_{int})	2547 (0.0758)	2020 (0.0867)	2081 (0.0687)
Reflns used in refinement, *n*	2547	2020	2081
L.S. parameters, *p*	133	116	117
No. of restraints, *r*	25	10	20
Completeness	0.996	0.994	1.00
$R1(F)^a$ $I > 2\sigma(I)$	0.0847	0.0901	0.1215
$wR2(F^2)^a$, all data	0.2195	0.2385	0.2883
$S(F^2)^a$, all data	1.100	1.026	1.164

[a] $R1(F) = \sum(|F_o| - |F_c|)/\sum|F_o|$; $wR2(F^2) = \sqrt{\sum w(F_o{}^2 - F_c{}^2)^2/\sum wF_o{}^4}$; $S(F^2) = \sqrt{\sum w(F_o{}^2 - F_c{}^2)^2/(n+r-p)}$.

[b] Density and adsorption coefficient are calculated using both framework atoms and cations even if the cations are unable to be modelled crystallographically

	In situ heating of **SHF-62-$CHCl_3$ (C5.2)**	*In situ* heating of **SHF-62-$CHCl_3$ (C6.1)**	*In situ* heating of **SHF-62-$CHCl_3$ (C6.2)**
Crystal Habit	Octahedron	Octahedron	Octahedron
Crystal Colour	Brown	Brown	Brown
Crystal Size (mm)	0.19 × 0.19 × 0.12	0.07 x 0.07 x 0.05	0.07 × 0.07 × 0.05
Crystal System	Orthorhombic	Orthorhombic	Orthorhombic
Space Group, Z	*Fddd*, 16	*Fddd*, 16	*Fddd*, 16
a (Å)	14.1740(10)	14.5562(2)	14.0557(8)
b (Å)	26.4532(17)	27.5448(4)	26.3239(10)
c (Å)	32.2074(18)	31.3490(3)	32.6166(10)
α (°)	90	90	90
β (°)	90	90	90
γ (°)	90	90	90
V (Å^3)	12076.1(13)	12569.3(3)	12068.2(9)
Radiation	Cu-K_α (λ = 1.54178 Å)	Sync (λ = 0.6889 Å)	Sync (λ = 0.6889 Å)
Density (g cm^{-3})[b]	1.327	1.275	1.328
Temperature (K)	298	298	298
μ (mm^{-1})	6.650	0.738	0.766
2θ Range (°)	7.59 to 117.86	3.316 to 58.944	4.83 to 51.006
Reflns collected	11563	37184	24421
Independent reflns (R_{int})	2155 (0.1307)	4795 (0.0391)	3088 (0.0528)
Reflns used in refinement, n	2155	4795	3088
L.S. parameters, p	125	145	130
No. of restraints, r	26	6	17
Completeness	1.00	1.00	1.00
$R1(F)$[a] $I > 2\sigma(I)$	0.0975	0.0383	0.0831
$wR2(F^2)$[a], all data	0.2424	0.1230	0.2704
$S(F^2)$[a], all data	1.109	1.007	1.114

[a] $R1(F) = \sum(|F_o| - |F_c|)/\sum|F_o|$; $wR2(F^2) = \sqrt{\sum w(F_o^2 - F_c^2)^2/\sum wF_o^4}$; $S(F^2) = \sqrt{\sum w(F_o^2 - F_c^2)^2/(n+r-p)}$.

[b] Density and adsorption coefficient are calculated using both framework atoms and cations even if the cations are unable to be modelled crystallographically

	In situ heating of **SHF-62-$CHCl_3$ (C7.1)**	*In situ* heating of **SHF-62-$CHCl_3$ (C7.2)**
Crystal Habit	Octahedron	Octahedron
Crystal Colour	Brown	Brown
Crystal Size (mm)	0.06 × 0.06 × 0.04	0.06 × 0.06 × 0.04
Crystal System	Orthorhombic	Orthorhombic
Space Group, Z	*Fddd*, 16	*Fddd*, 16
a (Å)	14.5486(2)	14.4748(2)
b (Å)	27.4966(4)	27.1714(5)
c (Å)	31.3509(4)	31.6622(5)
α (°)	90	90
β (°)	90	90
γ (°)	90	90
V (Å^3)	12541.5(3)	12452.8(3)
Radiation	Sync (λ = 0.6889 Å)	Sync (λ = 0.6889 Å)
Density (g cm^{-3})[b]	1.278	1.287
Temperature (K)	298	298
μ (mm^{-1})[c]	0.737	0.742
2θ Range (°)	3.318 to 55.478	3.332 to 55.474
Reflns collected	32574	32562
Independent reflns (R_{int})	4051 (0.0421)	4022 (0.0404)
Reflns used in refinement, n	40451	4022
L.S. parameters, p	145	143
No. of restraints, r	26	28
Completeness	1.00	1.00
$R1(F)$[a] $I > 2\sigma(I)$	0.0418	0.0460
$wR2(F^2)$[a], all data	0.1335	0.1485
$S(F^2)$[a], all data	1.094	1.077

[a] $R1(F) = \sum(|F_o| - |F_c|)/\sum|F_o|$; $wR2(F^2) = \sqrt{\sum w(F_o{}^2 - F_c{}^2)^2/\sum wF_o{}^4}$; $S(F^2) = \sqrt{\sum w(F_o{}^2 - F_c{}^2)^2/(n + r - p)}$.

[b] Densities are calculated using only framework atoms and cations, and do not include guest molecules.

[c] Adsorption coefficients are calculated using only framework atoms and cations, and do not include guest molecules.

3.2 Crystallographic studies of SHF-62-DMF following *in situ* heating

Crystals used for *in situ* heating were selected while immersed in the mother liquor and one face of the crystal glued to a glass fibre while the crystal was still covered in a thin layer of residual solvent. Care was taken to avoid coating the entire crystal in adhesive. The crystal was rapidly transferred to the diffractometer and situated in the nitrogen stream of the Cryostream device at 298 K. The glue was left to dry for 15 mins before any data were collected. Heating was carried out *in situ* using the Cryostream device. Two different heating experiments (**D1** & **D2**) were carried out on two separate crystals of **SHF-62-DMF**. A ramp rate of 4 K/min was used to raise the temperature, which was held at the final value for a specified period of time (see Table S4) before cooling at the same ramp rate. Intensity data collections were recorded using a laboratory diffractometer, as described in section 1.12, using the Cryostream device to maintain the temperature at 298 K under the nitrogen stream. The unit cell parameters were obtained by analysing reflections with $I/\sigma > 10$ from four sets of 10 ^{o} omega scans with 0.5 ^{o} slicing. A full data collection was recorded at the end of study **D1** due to the sample maintaining a high level of crystallinity (**D1.3**). Table S7 details the heating study parameters and the resulting cell parameters. Details of the full data collection **D1.3** are shown in Table S8.

Table S7. Parameters used and unit cell parameters determined for crystallographic studies of activation (desolvation) of **SHF-62-DMF** by *in situ* heating. All data were collected at 298 K.

Heating Study	Temp heated	Hold time	Data Set Code	*a* (Å)	*b* (Å)	*c* (Å)	*V* (Å^3)
**D1**	25 ^{o}C	Start	**D1.1**	14.715(4)	26.092(9)	32.283(7)	12395(6)
	150 ^{o}C	5 mins	**D1.2**	14.497(8)	25.74(2)	32.58(1)	12159(11)
	150 ^{o}C	15 mins	**D1.3**	13.8941(14)	25.686(2)	32.852(3)	11724(2)
**D2**	25 ^{o}C	Start	**D2.1**	14.82(2)	26.34(9)	32.09(3)	12520(20)
	100 ^{o}C	5 mins	**D2.2**	14.460(5)	25.824(6)	32.518(6)	12143(5)
	100 ^{o}C	10 mins	**D2.3**	14.172(8)	25.756(8)	32.671(8)	11925(8)
	100 ^{o}C	15 mins	**D2.4**	13.982(9)	25.709(9)	32.776(10)	11782(9)
	100 ^{o}C	30 mins	**D2.5**	13.760(7)	25.665(7)	32.869(8)	11608(7)
	150 ^{o}C	5 mins	**D2.6**	13.641(5)	25.677(6)	32.864(7)	11511(6)
	150 ^{o}C	30 mins	**D2.7**	13.652(5)	25.671(7)	32.831(6)	11506(5)

3.3 Crystallographic studies of SHF-62-DMF following *ex situ* heating

Crystals of **SHF-62-DMF** were transferred from the DMF solvent to a microscope slide and left until the DMF had evaporated. The crystals were then heated in a temperature-controlled oven at 150 ^{o}C for 15 minutes. After treatment the crystals were covered in a perfluoropolyether oil and mounted on a goniometer head. X-Ray diffraction data were recorded at 100 K under a cold nitrogen stream using a laboratory diffractometer , as described in section 1.12. The crystal retained most of its crystallinity, enabling detailed structural information to be obtained (**E1**). Full crystallographic information for the structure is listed in Table S8.

Table S8. Data collection, structure solution and refinement parameters for crystal structures during activation (solvent removal) studies on **SHF-62-DMF**

	In situ heating of **SHF-62-DMF (D1.3)**	*Ex situ* heating of **SHF-62-DMF (E1)**
Crystal Habit	Octahedron	Octahedron
Crystal Colour	Brown	Brown
Crystal Size (mm)	0.25 × 0.20 × 0.12	0.25 × 0.25 × 0.2
Crystal System	Orthorhombic	Orthorhombic
Space Group, Z	*Fddd*, 16	*Fddd*, 16
a (Å)	13.8941(14)	13.3875(6)
b (Å)	25.686(2)	25.0220(8)
c (Å)	32.852(3)	33.4491(7)
α (°)	90	90
β (°)	90	90
γ (°)	90	90
V (Å^3)	11724.3(19)	11204.8(6)
Radiation	Cu-K$_\alpha$ (λ = 1.54178 Å)	Cu-K$_\alpha$ (λ = 1.54178 Å)
Density (g cm^{-3})[b]	1.367	1.430
Temperature (K)	298	100
μ (mm^{-1})[c]	6.899	7.218
2θ Range (°)	7.72 to 101.16	7.94 to 133.15
Reflns collected	8399	10520
Independent reflns (R_{int})	1525 (0.0532)	2434 (0.0507)
Reflns used in refinement, n	1525	2434
L.S. parameters, p	117	132
No. of restraints, r	44	23
Completeness	0.985	0.979
$R1(F)$[a] $I > 2\sigma(I)$	0.0965	0.0895
$wR2(F^2)$[a], all data	0.2984	0.2415
$S(F^2)$[a], all data	1.159	1.137

[a] $R1(F) = \sum(|F_o| - |F_c|)/\sum|F_o|$; $wR2(F^2) = \sqrt{\sum w(F_o^2 - F_c^2)^2/\sum wF_o^4}$; $S(F^2) = \sqrt{\sum w(F_o^2 - F_c^2)^2/(n + r - p)}$.

[b] Densities are calculated using only framework atoms and cations, and do not include guest molecules.

[c] Adsorption coefficients are calculated using only framework atoms and cations, and do not include guest molecules.

4. Additional Graphical Representation of Breathing Behaviour

4.1 Activation-flip modelled from single-crystal structures

The single-crystal structures determined during the studies of activation of **SHF-62-CHC**l$_3$ and **SHF-62-DMF** show that there is a gradual response to *a*-axis contraction as solvent is removed, which results in the 150 ° activation-flip of a proportion of the methylamidobenzendicaboxylate (BDC-NHC(O)Me) ligands. The activation flip is first detected at $a \approx 14.2$ Å and reaches the maximum extent (approx. 50% of ligands flipped) as the the pores contract further to $a < 13.7$ Å (Figure S13).

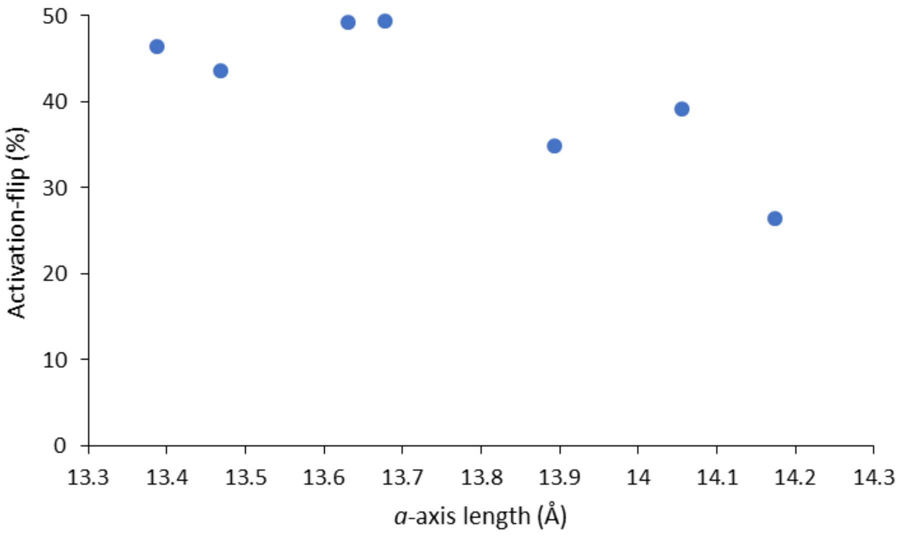

Figure S13. Percentage of ligands undergoing activation-flip as a function of *a*-axis length (pore length) during activation of **SHF-62-solvent.** (Range of values is 26(1) – 49(1) %.)

4.2 Activation-flip modelled from single crystal X-ray diffraction: comparison of SHF-62-CHCl$_3$ with SHF-62-DMF

The series of single-crystal X-ray diffraction studies used to determine the crystals structure and/or unit-cell dimensions presented in Figure 2g and 2h were used to establish the regions in which the activation-flip occurs in terms of pore widths (*b*- and *c*-axes) and pore length (*a*-axis). Separation of these studies based upon which pore solvent is being removed (Figure S14) confirms that the region in which the activation-flip occurs lies in a narrow range of dimensions of pore length (Figure S14b and S14d), but suggests that the activation-flip may occur at a narrower-pore stage for **SHF-62-DMF** (Figure S14c) than for **SHF-62-CHCl$_3$** (Figure S14a).

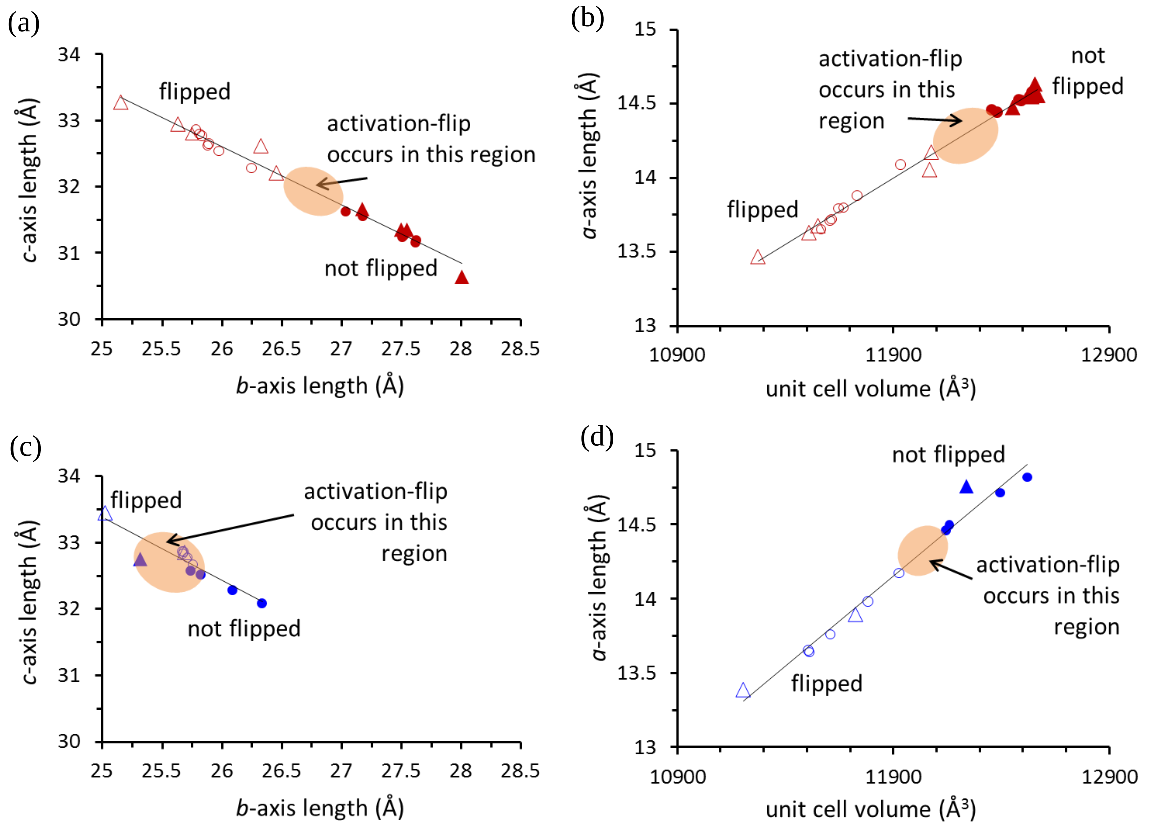

Figure S14. Detailed representation of Figures 2g and 2h as a function of activation solvent, showing structural changes in **SHF-62-CHCl$_3$** (red) and **SHF-62-DMF** (blue) and highlighting the region in which the ligand ring-flip (activation-flip) occurs, enabling substantial contraction of the pore length and correspondingly of the *a*-axis. Open symbols represent flipped-ring stuctures (i.e. 26-50% ring flipping is modelled); triangles represent full structure determination; circles represent unit-cell determination. (Note: for unit-cell determinations, flipped or non-flipped status is inferred from *a*-axis value)

5. References:

S1. E. J. Carrington, C. A. McAnally, A. J. Fletcher, S. P. Thompson, M. Warren, L. Brammer, *Nat. Chem.* **2017**, *9*, 882-889.

S2. Bruker *APEX2*. Bruker AXS Inc., Madison, Wisconsin, USA, 2007

S3. Rigaku *CrysAlis Pro*. Rigaku Oxford Diffraction, Oxfordshire, UK, 2016

S4. (a) *SADABS*, empirical adsorption correction program,[S4b] based on the method of Blessing.[S4c] (b) L. Krause, R. Herbst-Irmer, G. M. Sheldrick and D. Stalke, *J. Appl. Cryst.* **2015**, *48*, 3–10. (c) R. H. Blessing, *Acta Crystallogr.* **1995**, *A51*, 33–38.

S5. H. Nowell, S. A. Barnett, K. E. Christensen, S. J. Teat and D. R. Allan, J. *Synchrotron Radiat.* **2012**, *19*, 435–441.

S6. G. Winter, D. G. Waterman, J. M. Parkhurst, A. S. Brewster, R. J. Gildea, M. Gerstel, L. Fuentes-Montero, M. Vollmar, T. Michels-Clark, I. D. Young, N. K. Sauter, G. Evans, *Acta. Crystallogr.* **2018**, D74, 85-97.

S7. G. M. Sheldrick, *Acta Crystallogr.* **2015**, *C71*, 3–8.

S8. O. V. Dolomanov, L. J. Bourhis, R. J. Gildea, J. A. K. Howard and H. Puschmann, *J. Appl. Cryst.* **2009**, *42*, 339–341.

S9. A. L. Spek, Acta Crystallogr. **2015**, *C71*, 9–18.

S10. A. L. Spek, *Acta Crystallogr.* **2009**, *D65*, 148–55.

S11. S. P. Thompson, J. E. Parker, J. Potter, T. P. Hill, A. Birt, T. M. Cobb, F. Yuan and C. C. Tang, *Rev. Sci. Instrum.* **2009**, *80*, 075107.

S12. S. P. Thompson, J. E. Parker, J. Marchal, J. Potter, A. Birt, F. Yuan, R. D. Fearn, A. R. Lennie, S. R. Street and C. C. Tang, *J. Synchrotron Radiat.* **2011**, *18*, 637–648.

S13. G. S. Pawley, *J. Appl. Cryst.* **1981**, *14*, 357–361.

S14. H. M. Rietveld, *J. Appl. Cryst.* **1969**, *2*, 65–71.

S15. (a) A. A. Coelho, *TOPAS Academic Version 4.1, 2007, see http//www.topas-academic.net.* (b) A. A. Coelho, *J. Appl. Cryst.*, **2018**, *51*, 210–218; (c) A. A. Coelho, J. Evans, I. Evans, A. Kern and S. Parsons, *Powder Diffr.*, **2011**, *26*, S22–S25.

S16. A. B. Pangborn, M. A. Giardello, R. H. Grubbs, R. K. Rosen and F. J. Timmers, *Organometallics* **1996**, *15*, 1518–1520.
